# Mapping traditional Chinese medicine-related complementary interventions for cerebral palsy rehabilitation: a bibliometric and visualization analysis

**DOI:** 10.3389/fneur.2026.1882028

**Published:** 2026-08-26

**Authors:** Zhuoluo Zhou, Ting Wang, Xiaoye Li, Bowen Deng, Houjun Zhang, Yu Jiang, Yuxin Xue, Lin Xu, Xiaohong Mu, Jinyu Li

**Affiliations:** 1 Dongzhimen Hospital, Beijing University of Chinese Medicine, Beijing, China; 2 Beijing Hospital of Traditional Chinese Medicine, Beijing, China

**Keywords:** acupuncture, bibliometric analysis, cerebral palsy, rehabilitation, scientometric mapping, traditional Chinese medicine

## Abstract

**Purpose:**

This bibliometric and scientometric study mapped the development, collaboration structure, thematic organization, and temporal evolution of Chinese- and English-language research on traditional Chinese medicine (TCM)-related interventions for cerebral palsy (CP).

**Methods:**

Publications were retrieved from Web of Science Core Collection, Scopus, PubMed, Embase, China National Knowledge Infrastructure, Wanfang Data, and the Chinese Science and Technology Journal Database from inception to July 20, 2026. Eligible peer-reviewed articles and reviews were analyzed separately by language. Excel supported descriptive analysis, VOSviewer keyword co-occurrence and overlay mapping, CiteSpace keyword burst detection, and Python with NetworkX coauthorship analysis. Parameter sensitivity analyses and an author-developed integrative thematic synthesis were also performed.

**Results:**

The final corpora included 3,225 Chinese-language and 230 English-language publications. Chinese-language output rose markedly after the late 2000s and peaked in 2020, whereas English-language output remained smaller and more variable. The English-language coauthorship network was more connected and had a higher multi-author publication rate. At a minimum occurrence threshold of five, 179 of 2,115 standardized Chinese keywords and 70 of 955 standardized English keywords were retained. Five research domains were identified: clinical rehabilitation and functional outcomes; spasticity and symptom-oriented management; TCM intervention modalities; clinical research design and evidence synthesis; and experimental and mechanistic research. Temporal analyses indicated a broadening from general clinical applications toward function-oriented assessment, evidence synthesis, and mechanistic investigation. Sensitivity analyses supported the broad thematic structure, although several recent burst signals were parameter-sensitive.

**Conclusion:**

The corpora showed distinct publication and collaboration patterns but overlapping thematic structures. These findings map the literature’s organization and evolution but do not establish efficacy, safety, or certainty of evidence. Priorities include prospectively registered multicenter studies, standardized intervention reporting, a core outcome set, systematic safety assessment, and clinically anchored mechanistic research.

## 1 Introduction

Cerebral palsy (CP) comprises a group of permanent disorders of movement and posture resulting from non-progressive disturbances in the developing fetal or infant brain (1). It is one of the most common causes of childhood physical disability and is frequently accompanied by sensory, cognitive, communication, behavioral, and musculoskeletal impairments (2). Children with CP usually require long-term rehabilitation, and place a considerable burden on families and healthcare systems worldwide (3).

Current management of CP is individualized and commonly combines physical and occupational therapy, speech and language therapy, orthotic management, pharmacological treatment for spasticity, and selected orthopedic or neurosurgical procedures (4). Although these approaches can improve specific aspects of motor function, mobility, participation, and symptom control, many children continue to experience persistent functional limitations, secondary complications, and restricted participation in daily life. Access to comprehensive multidisciplinary rehabilitation also remains uneven, particularly in resource-constrained settings (5). These unmet needs have contributed to continued interest in adjunctive and integrative rehabilitation approaches.

Traditional Chinese medicine (TCM)-related interventions, including acupuncture, electroacupuncture, scalp acupuncture, moxibustion, tuina or massage, Chinese herbal medicine, and combinations of these modalities with conventional rehabilitation, have been investigated in children with CP, particularly in China and other East Asian settings (6). Clinical studies and randomized controlled trials have examined outcomes such as gross motor function, muscle tone, activities of daily living, pain, and quality of life (7, 8). Experimental and neuroimaging studies have also explored potential biological processes involving neuroplasticity, neuroprotection, inflammatory regulation, apoptosis, and white matter injury (9, 10). Although these studies span clinical rehabilitation, evidence synthesis, neuroimaging, and preclinical investigation, the available evidence base remains relatively limited and methodologically uneven, with marked variation in intervention content and dose, participant age, CP subtype and severity, comparator selection, outcome measurement, and study quality, together with geographically concentrated samples and an incompletely established relationship between proposed mechanisms and clinical outcomes.

Existing systematic reviews and meta-analyses have mainly evaluated selected TCM-related interventions or specific clinical outcomes in children with CP (6, 11). Although these reviews are important for summarizing intervention-specific evidence, they do not fully characterize how the broader research field has developed, how research activity is distributed among countries, institutions, journals, and author groups, or how clinical, methodological, and mechanism-oriented themes are connected. Previous bibliometric studies have characterized the broader CP literature and selected subdomains, including overall publication patterns, major contributors, collaboration networks, research hotspots, and highly cited neuroimaging studies (12, 13). However, these analyses did not specifically isolate TCM-related complementary interventions within CP rehabilitation. This distinction is important because intervention-focused reviews have reported a limited, heterogeneous, and geographically concentrated primary evidence base, while broader CP research initiatives have emphasized the need for coordinated international research infrastructure capable of delivering more timely and methodologically robust studies. Taken together, these observations leave several field-level questions unresolved: whether research capacity in this subfield is concentrated within a small number of countries, institutions, and author groups; whether collaboration extends beyond local or national networks; and whether clinical rehabilitation, evidence synthesis, and mechanism-oriented research have developed as connected or disproportionate strands.

To address these gaps, the present study used a multilingual, multi-database retrieval framework incorporating the Web of Science Core Collection, Scopus, PubMed, Embase, China National Knowledge Infrastructure, Wanfang Data, and the Chinese Science and Technology Journal Database. Combining international and Chinese databases broadens the coverage of clinical, evidence-synthesis, and mechanism-oriented research and reduces the database- and language-related selection bias associated with reliance on English-language multidisciplinary indexes alone. After systematic deduplication and cross-database standardization, complementary bibliometric analyses were integrated to examine publication patterns, collaboration structures, thematic organization, and temporal changes, and to assess the consistency of findings across analytical approaches. This design improves the representativeness and robustness of the mapped research landscape without treating bibliometric prominence as evidence of clinical efficacy or evidence quality. A dedicated mapping of this subfield is therefore needed to complement, rather than duplicate, intervention-specific evidence reviews.

Specifically, this study addressed three research questions: (1) How do publication trajectories, geographical and institutional contributions, journal distribution, and coauthorship structures differ between the Chinese- and English-language corpora? (2) What thematic structures and temporal patterns are identified within each corpus through keyword co-occurrence, overlay visualization, and burst analysis? (3) Which broader research domains are consistently supported across the analytical outputs, and what methodological priorities emerge from the mapped research landscape?

Accordingly, the contribution of this study lies in three linked features. First, it applies a multilingual, multi-database framework to map a clinically relevant but bibliographically fragmented field, thereby improving the representation of research published in both international and Chinese sources. Second, it moves beyond productivity rankings by examining collaboration structure and triangulating thematic patterns across co-occurrence, overlay, burst, and coauthorship analyses. Third, it synthesizes the convergent findings into an author-derived thematic research landscape to identify areas of research concentration, thematic disconnection, and methodological priority, while explicitly avoiding inferences regarding clinical efficacy, safety, or certainty of evidence.

## 2 Methods

### 2.1 Data sources and search strategy

A comprehensive multilingual literature search was conducted in the Web of Science Core Collection (WoSCC), Scopus, PubMed, Embase, China National Knowledge Infrastructure (CNKI), Wanfang Data, and the Chinese Science and Technology Journal Database (VIP) from database inception to July 20, 2026. All retrieved records were entered into a common deduplication and eligibility-screening workflow.

The information sources were selected for complementary purposes. WoSCC and Scopus were retained because they provide broad multidisciplinary coverage and structured bibliographic and citation metadata suitable for research mapping. PubMed and Embase were included to strengthen the retrieval of biomedical, pediatric, neurological, rehabilitation, and pharmacological literature. PubMed primarily supports the retrieval of biomedical and life-sciences publications, whereas Embase provides a dedicated biomedical indexing system with extensive clinical and pharmacological coverage. CNKI, Wanfang Data, and VIP were included to capture Chinese-language studies on TCM, acupuncture, tuina, Chinese herbal medicine, and integrated rehabilitation that may be underrepresented in international databases.

The search strategy combined terms related to cerebral palsy with terms related to TCM-associated interventions. Cerebral palsy-related terms included “cerebral palsy,” “spastic cerebral palsy,” “infantile cerebral palsy,” and their Chinese equivalents. Intervention-related terms included “traditional Chinese medicine,” “Chinese medicine,” “Chinese herbal medicine,” “herbal formula*,” “acupuncture,” “electroacupuncture,” “scalp acupuncture,” “moxibustion,” “tuina,” “massage,” and their corresponding Chinese terms and spelling variants. Controlled vocabulary was combined with free-text terms where available, including Medical Subject Headings in PubMed and Emtree terms in Embase. Database-specific field codes and syntax were used for each source. The complete search strategy, platform, search date, and number of records retrieved from each database are provided in Supplementary Table S1. No publication-year restriction was applied, and publications in English or Chinese were eligible for screening.

Because the included databases differ in journal coverage, indexing practices, controlled vocabularies, and the completeness of bibliographic and citation fields, the expanded search was expected to reduce—but not eliminate—database and language-related selection bias. Potential inconsistencies arising from duplicate records, bilingual titles and keywords, author and institution name variants, and database-specific metadata were addressed through prespecified deduplication and standardization procedures described in Section 2.2. Citation counts from different databases were not summed directly; the sources and procedures used for citation-dependent analyses are described in Section 2.3.

### 2.2 Eligibility criteria and study selection

Publications were eligible if they met all of the following criteria:

(1) they were full-length, peer-reviewed original research articles or review articles, including systematic reviews, meta-analyses, scoping reviews, and narrative reviews; (2) cerebral palsy (CP) was the primary target condition; (3) at least one traditional Chinese medicine (TCM)-related intervention was a central component of the research question, intervention, comparator, or review objective; and (4) they were published in English or Chinese and contained sufficient information for eligibility assessment and bibliometric analysis.

For human studies, participants were required to have an explicit diagnosis of CP. Studies involving mixed pediatric neurological populations were included only when CP was a prespecified study population and CP-specific findings could be identified separately. Studies in which CP was mentioned only in the background, discussion, or a general list of neurological conditions were excluded. Full-length case reports and case series were eligible when the reported patient or patients had CP and a qualifying TCM-related intervention was central to the report.

Preclinical studies were included only when the authors explicitly defined the experimental model as a model of CP or spastic CP and evaluated a qualifying TCM-related intervention in relation to CP-relevant outcomes. Studies using models of neonatal hypoxic–ischemic brain injury, cerebral ischemia, white matter injury, or other neurological conditions were excluded when these models were presented only as general models of neurological injury and were not explicitly established or interpreted as CP models. Reviews involving multiple pediatric disorders or complementary interventions were included only when CP was an explicit target population and CP-specific findings were separately identifiable.

Eligible TCM-related interventions included Chinese herbal medicine, Chinese patent or proprietary medicines, acupuncture, electroacupuncture, scalp acupuncture, auricular acupuncture, moxibustion, tuina, Chinese therapeutic massage, cupping, gua sha, acupoint application or herbal patching, and integrated TCM–conventional rehabilitation. Generic herbal medicine, massage, scraping, topical patches, or dry needling were not considered TCM-related unless the publication explicitly identified the intervention as a Chinese medicine modality or described a qualifying TCM-specific technique. Publications that mentioned CP or a TCM-related intervention only incidentally were excluded.

Conference abstracts, conference proceedings without a full peer-reviewed article, editorials, commentaries, letters, corrections, retraction notices, book chapters, dissertations, preprints, trial-registration records, and study protocols without outcome data were excluded. Complete peer-reviewed Early Access or articles-in-press publications were eligible. When both an Early Access record and the final version of record were retrieved, they were treated as one publication and the final version was retained. Articles formally retracted before the final data-verification date were excluded, and corrections or errata were not counted as independent publications.

Records were initially screened within each database using the prespecified eligibility criteria. Records retained after final eligibility assessment were subsequently merged within each language-specific corpus, after which cross-database duplicates were identified and removed. Bibliographic duplicates were identified hierarchically using DOI, PubMed ID, database accession numbers, normalized titles, authors, publication year, journal, and page or article numbers. Chinese and English title variants were manually cross-checked when necessary.

Two reviewers independently screened titles and abstracts using the prespecified eligibility criteria. Potentially eligible records subsequently underwent independent full-text assessment. Reasons for full-text exclusion were recorded using predefined categories. Disagreements were resolved through discussion, and unresolved cases were adjudicated by a third reviewer.

Before the final analytical corpus was established, all eligible publications underwent a final identity and version check. Early Access, article-in-press, and final versions referring to the same publication were consolidated, with the final version of record retained. Residual duplicate records identified through bilingual title matching or incomplete database metadata were removed. Distinct peer-reviewed publications arising from the same underlying study were retained as separate publications when each met the eligibility criteria, but were linked using a common study identifier where the relationship could be established.

### 2.3 Data extraction and bibliographic standardization

Following study selection, two reviewers independently checked and standardized the bibliographic records using prespecified cleaning rules. Before formal cleaning, the reviewers calibrated the procedure on a sample of included records. The original database exports were retained, and all corrections, exclusions, and term-merging decisions were documented.

Bibliographic fields used in the descriptive and visualization analyses included title, authors, affiliations, journal, publication year, document type, language, keywords, indexing terms, cited references, and citation information where available. Author, institution, and journal names were standardized only when necessary for descriptive tabulation. Journal titles were checked using ISSN and official journal names, and obvious spelling or formatting variants in institutional and author names were corrected using the original affiliations and other available bibliographic information. Ambiguous author identities were not merged.

Keyword cleaning was performed separately for the Chinese- and English-language datasets. Spelling and hyphenation variants, singular and plural forms, unambiguous abbreviations, and genuine synonyms were merged using a prespecified keyword-merging thesaurus. Conceptually distinct interventions, populations, outcomes, mechanisms, and study designs were retained as separate terms. Database labels, document-type terms, generic indexing terms, and other terms without substantive thematic meaning were removed using a predefined exclusion thesaurus. No keywords were manually removed after visualization solely to improve the appearance of the maps.

Data cleaning was independently performed by two reviewers. Disagreements regarding record retention, bibliographic correction, keyword merging, or term exclusion were resolved through discussion and consensus; unresolved disagreements were adjudicated by a third reviewer.

The original database search records, the deduplicated datasets, the Chinese and English keyword-merging and exclusion thesaurus are provided in the Supplementary material, subject to database licensing restrictions.

### 2.4 Bibliometric and visualization analysis

Descriptive bibliometric indicators were summarized using Microsoft Excel 2019. Annual publication output and the distributions of document type, language, journal, author, institution, intervention category, and study-design category were presented in tables or descriptive charts. Publications indexed in 2026 were included in the descriptive, annual-trend, keyword co-occurrence, and overlay analyses, but annual counts for 2026 were treated as incomplete. The 2026 records were excluded from the CiteSpace temporal and burst analyses.

VOSviewer is widely used in bibliometric and scientometric studies due to its ability to handle large datasets, automatically detect clusters, and produce intuitive visualizations that reveal structural patterns, research hotspots, and collaboration relationships (14, 15). VOSviewer version 1.6.20 was used exclusively to construct keyword co-occurrence networks and overlay visualizations. The Chinese- and English-language datasets were analyzed separately. Keyword analyses were based on the cleaned keyword data obtained after application of the keyword-merging and exclusion thesauri described in Section 2.3.

For keyword co-occurrence analysis, the type of analysis was co-occurrence and the unit of analysis was keywords. The counting method was set to full counting, and link strengths were normalized using the association-strength method. The minimum occurrence threshold was set at 5 for the Chinese dataset and the same for the English dataset. All cleaned keywords meeting the corresponding threshold were included; no additional nodes were selected or removed according to their network position.

The VOSviewer layout parameters were set to attraction = 10 and repulsion = 6 for Chinese dataset, whereas attraction = 2 and repulsion = 0 for English dataset. The minimum cluster size was set to 10 for the Chinese network and 1 for the English network, reflecting differences in network size and density. Node size represented keyword occurrence frequency, link thickness represented co-occurrence strength, and node color represented cluster membership. Clusters were generated automatically by the VOSviewer clustering algorithm; cluster membership was not assigned manually. Descriptive thematic labels were subsequently assigned by examining the keywords with the highest occurrence frequencies and total link strengths within each cluster and evaluating the semantic coherence of the remaining terms. These labels were used only to summarize the predominant content of each cluster and did not affect node membership or network structure.

Overlay visualizations were generated using the same records, keyword set, occurrence threshold, counting method, and network structure as the corresponding co-occurrence maps. In the overlay visualizations, node color represented the average publication year of the publications in which each keyword occurred.

CiteSpace version 6.4.2 was used to examine temporal keyword patterns and detect burst keywords separately in the Chinese- and English-language datasets (16). The analysis periods were 1989–2025 for the Chinese dataset and 2005–2025 for the English dataset, with 1 year per time slice. Although a small number of records were published before these starting years, they contained no keywords or an insufficient number of indexable keywords to support meaningful annual keyword analysis. These records were therefore not included in the keyword-based temporal and burst analyses.

The node type was set to Keyword, and nodes were selected within each annual time slice using the g-index with k = 25. No network-pruning option was applied. Keyword bursts were detected using Kleinberg’s burst-detection algorithm with two burst states and an alpha ratio of 2.0. For the Chinese-language corpus, *γ* was set to 1.0 and the minimum burst duration to 2 years. For the English-language corpus, the principal analysis used γ = 0.5 and a minimum burst duration of 1 year. The alternative English-language settings are described in Section 2.5. Records published in 2026 were excluded from the temporal and burst analyses because 2026 represented an incomplete publication year.

### 2.5 Parameter sensitivity analyses

To assess the robustness of the keyword-mapping results, parameter sensitivity analyses were conducted separately for the Chinese- and English-language corpora. For the VOSviewer analyses, alternative minimum-occurrence thresholds were examined while the dataset, thesaurus file, keyword field, full-counting method, association-strength normalization, clustering resolution, and layout parameters were held constant. The resulting networks were compared in terms of the number of retained keywords, links, total link strength, cluster composition, leading keywords, and overall thematic interpretation.

For the Chinese-language corpus, minimum-occurrence thresholds of 3, 5, and 10 were examined, with 5 used in the principal analysis. For the English-language corpus, thresholds of 3, 5, and 7 were examined, with 5 used in the principal analysis. For the Chinese-language network, the minimum cluster size was fixed at 10 when the occurrence threshold was varied. The English-language network used a minimum cluster size of 1. All other data-processing, normalization, clustering-resolution, and layout parameters were held constant.

For the CiteSpace burst analysis of the English-language corpus, the principal analysis used *γ* = 0.5 and a minimum burst duration of 1 year. Two more conservative settings were examined: γ = 0.6 with a minimum duration of 1 year and *γ* = 0.6 with a minimum duration of 2 years. All other parameters were held constant. The Chinese-language burst analysis used γ = 1.0 and a minimum burst duration of 2 years. Sensitivity analyses were used to evaluate parameter-related variation and were not used to select results according to their agreement with expected findings.

### 2.6 Author productivity and coauthorship analysis

Author productivity and coauthorship patterns were analyzed separately for the Chinese- and English-language corpora. Records with available author information were included in the author-level analysis. Author productivity was calculated using full counting, whereby each listed author received one publication count for each coauthored record. The 10 authors with the largest numbers of publications were identified for each corpus.

An undirected weighted coauthorship network was constructed for each corpus. Authors were represented as nodes, and an edge was established between two authors when they had coauthored at least one publication. Edge weight was defined as the number of publications jointly authored by the corresponding author pair. Repeated author pairs were aggregated, and self-loops were excluded. Network-level metrics were calculated using the complete, unthresholded coauthorship networks.

Network structure was characterized using the number of authors and coauthorship links, network density, number of connected components, size and proportion of the largest connected component, mean degree, average clustering coefficient, number of isolated authors, proportion of multi-author publications, and mean number of authors per publication. Degree centrality was calculated in the complete network and normalized by the maximum possible degree. Betweenness centrality was calculated within the largest connected component using normalized, unweighted shortest paths, with path endpoints excluded. Weighted collaboration strength was calculated as the sum of the weights of all coauthorship links associated with an author. The average clustering coefficient was calculated using the unweighted network.

Descriptive author statistics were compiled using Microsoft Excel 2019, and network metrics were calculated using Python version 3.13.5 and NetworkX version 3.6.1. Author names were harmonized for differences in punctuation, spacing, capitalization, and clearly identifiable spelling variants. Because the available bibliographic metadata did not support complete author-identity disambiguation, authors with identical names or initials could not always be distinguished reliably.

### 2.7 Integrative thematic synthesis

Following the keyword co-occurrence, overlay, and burst analyses, an integrative thematic synthesis was conducted to summarize the principal research domains represented across the two corpora. This framework was developed by the authors and was not generated automatically by VOSviewer or CiteSpace. Candidate domains were identified by jointly considering: (1) keywords with high occurrence frequencies and total link strengths within the software-generated co-occurrence clusters; (2) the temporal distribution of keywords in the overlay visualizations; and (3) keywords showing marked occurrence bursts in the CiteSpace analyses. Semantically related terms were grouped into broader descriptive domains. A domain was retained when it was supported by at least two of the three analytical outputs and represented a coherent research topic within the included literature. Two reviewers independently examined the candidate terms, assigned them to domains, and proposed descriptive domain labels. Disagreements were resolved through discussion and consensus. The resulting framework was intended to summarize thematic structure and temporal research emphasis and did not represent an assessment of study quality, clinical effectiveness, risk of bias, or level of evidence.

## 3 Results

### 3.1 Study selection

**Figure 1:**
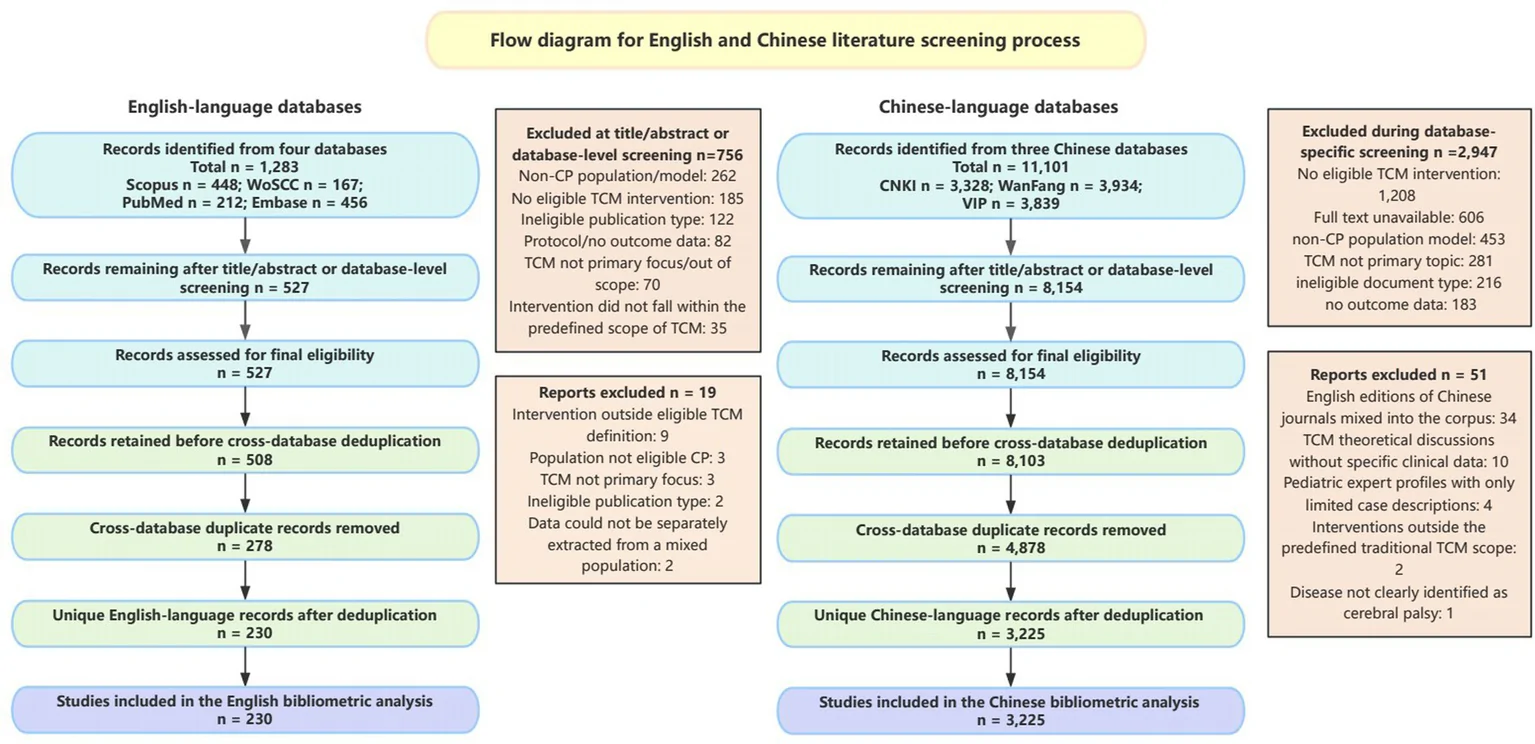
Flowchart of literature retrieval, screening, and study selection.

Database searches identified 11,101 Chinese-language records, including 3,328 from CNKI, 3,934 from Wanfang Data, and 3,839 from VIP, and 1,283 English-language records, including 448 from Scopus, 167 from WoSCC, 212 from PubMed, and 456 from Embase. Database-specific screening excluded 2,947 Chinese-language records and 756 English-language records. After final eligibility assessment, a further 51 Chinese-language records and 19 English-language records were excluded, leaving 8,103 and 508 records, respectively, before cross-database deduplication. Removal of 4,878 Chinese-language and 278 English-language duplicate records yielded final analytical corpora of 3,225 and 230 publications, respectively (Figure 1).

### 3.2 Annual publication trends

Annual publication trends were examined using the complete bibliographic corpora, comprising 3,225 records from the Chinese-language corpus and 230 records from the English-language corpus. All records with available publication-year information were included in the annual distribution. One record in the Chinese-language corpus lacked a valid publication year and could not be assigned to a specific year.

Publication activity in the Chinese-language corpus was sparse and intermittent during the early period, followed by a gradual increase from the mid-1990s onward. A more pronounced expansion occurred after the late 2000s, and annual output reached its highest level in 2020. Publication volume subsequently declined from 2021 onward. The lower output observed in 2026 should be interpreted cautiously because the year was incomplete at the time of data retrieval.

The English-language corpus showed a smaller and more fluctuating publication pattern. Early publication activity was sporadic, followed by a gradual increase after the mid-2000s. Annual output remained relatively limited but sustained during the most recent decade, with intermittent peaks rather than a continuous linear increase. The publication count for 2026 was also incomplete and was not interpreted as evidence of a decline.

Overall, the Chinese-language corpus showed a marked transition from limited early activity to substantial expansion, followed by a reduction after the 2020 peak. In contrast, the English-language corpus remained considerably smaller and exhibited a more variable pattern of sustained low-volume publication activity. Because of the substantial difference in corpus size, the two annual publication series were presented in separate panels and compared in terms of temporal patterns rather than absolute publication volume.

### 3.3 Countries/regions and institutions

In the English-language corpus, publication output was markedly concentrated in China (Table 1). China contributed 154 publications, accounting for 67.0% of the 230 records, followed by the United States with nine publications (3.9%). Hong Kong and South Korea each contributed five publications (2.2%), whereas Egypt, Israel, and Taiwan each contributed three publications (1.3%). Austria, the Czech Republic, Malaysia, Russia, and the United Kingdom each contributed two publications (0.9%). Overall, the leading countries and regions other than China contributed relatively small numbers of publications, indicating limited geographical diversification within the English-language literature.

**Table 1:** Leading contributing countries/regions in the English-language corpus.

Rank	Country/Region	Publications	Percentage of corpus, %
1	China	154	67.0
2	United States	9	3.9
3	Hong Kong	5	2.2
3	South Korea	5	2.2
5	Egypt	3	1.3
5	Israel	3	1.3
5	Taiwan	3	1.3
8	Austria	2	0.9
8	Czech Republic	2	0.9
8	Malaysia	2	0.9
8	Russia	2	0.9
8	United Kingdom	2	0.9

Within the Chinese-language corpus, publications were distributed across multiple provincial-level regions, although several regions contributed disproportionately large shares (Table 2). Henan ranked first with 575 publications, representing 17.8% of the 3,225 records, followed by Guangdong with 326 publications (10.1%) and Jiangsu with 187 publications (5.8%). Shandong, Hunan, Liaoning, and Hebei contributed 140 (4.3%), 136 (4.2%), 134 (4.2%), and 128 (4.0%) publications, respectively. Shaanxi contributed 121 publications (3.8%), while Beijing contributed 93 (2.9%). Guangxi and Jiangxi each contributed 92 publications (2.9%) and shared tenth place.

**Table 2:** Leading contributing provincial-level regions in the Chinese-language corpus.

Rank	Region	Publications	Percentage of corpus, %
1	Henan	575	17.8
2	Guangdong	326	10.1
3	Jiangsu	187	5.8
4	Shandong	140	4.3
5	Hunan	136	4.2
6	Liaoning	134	4.2
7	Hebei	128	4.0
8	Shanxi	121	3.8
9	Beijing	93	2.9
10	Guangxi	92	2.9
10	Jiangxi	92	2.9

Taken together, the English-language literature was strongly concentrated in publications involving institutions in China, with comparatively limited contributions from other countries and regions. The Chinese-language literature showed a broader domestic distribution, but publication activity was particularly concentrated in Henan and Guangdong, which together accounted for 27.9% of the corpus.

In the English-language corpus, Nanhai Maternity and Children’s Hospital Affiliated to Guangzhou University of Chinese Medicine ranked first with 14 publications, accounting for 6.1% of the 230 records (Table 3). Capital Medical University and Guangzhou University of Chinese Medicine each contributed six publications (2.6%), followed by Fudan University and Shanghai University of Traditional Chinese Medicine, with five publications each (2.2%). Beijing Children’s Hospital, the China Academy of Chinese Medical Sciences, Jinshan Hospital of Fudan University, Nanchang University, and Sichuan University each contributed four publications (1.7%).

**Table 3:** Top 10 institutions by publication output in the English-language corpus of research on Traditional Chinese Medicine-related interventions for cerebral palsy.

Rank	Institutions	Publications	Proportion
1	Nanhai Maternity and Children’s Hospital Affiliated to Guangzhou University of Chinese Medicine	14	6.1%
2	Capital Medical University	6	2.6%
3	Guangzhou University of Chinese Medicine	6	2.6%
4	Fudan University	5	2.2%
5	Shanghai University of Traditional Chinese Medicine	5	2.2%
6	Beijing Children’s Hospital	4	1.7%
7	China Academy of Chinese Medical Sciences	4	1.7%
8	Jinshan Hospital, Fudan University	4	1.7%
9	Nanchang University	4	1.7%
10	Sichuan University	4	1.7%

In the Chinese-language corpus, Nanhai Maternity and Children’s Hospital Affiliated to Guangzhou University of Chinese Medicine also ranked first, contributing 87 publications, or 2.7% of the 3,225 records (Table 4). Children’s Hospital Affiliated to Zhengzhou University ranked second with 85 publications (2.6%), followed by the First Affiliated Hospital of Henan University of Chinese Medicine with 58 publications (1.8%). Guangzhou University of Chinese Medicine and Shenyang Children’s Hospital each contributed 49 publications (1.5%), while Hunan Children’s Hospital contributed 46 publications (1.4%). The remaining leading institutions were the Second Affiliated Hospital of Henan University of Chinese Medicine (43 publications, 1.3%), the First Affiliated Hospital of Liaoning University of Traditional Chinese Medicine (39, 1.2%), the Third Affiliated Hospital of Zhengzhou University (37, 1.1%), and Xi’an Traditional Chinese Medicine Hospital of Encephalopathy (32, 1.0%).

**Table 4:** Top 10 institutions by publication output in the Chinese-language corpus of research on traditional Chinese medicine-related interventions for cerebral palsy.

Rank	Institutions	Publications	Proportion
1	Nanhai Maternity and Children’s Hospital Affiliated to Guangzhou University of Chinese Medicine	87	2.7%
2	Children’s Hospital Affiliated to Zhengzhou University (Henan Children’s Hospital/Zhengzhou Children’s Hospital)	85	2.6%
3	The First Affiliated Hospital of Henan University of Chinese Medicine	58	1.8%
4	Guangzhou University of Chinese Medicine	49	1.5%
5	Shenyang Children’s Hospital	49	1.5%
6	Hunan Children’s Hospital	46	1.4%
7	The Second Affiliated Hospital of Henan University of Chinese Medicine (Henan Provincial Hospital of Traditional Chinese Medicine)	43	1.3%
8	The First Affiliated Hospital of Liaoning University of Traditional Chinese Medicine	39	1.2%
9	The Third Affiliated Hospital of Zhengzhou University	37	1.1%
10	Xi’an Traditional Chinese Medicine Hospital of Encephalopathy	32	1.0%

Across the two language-specific corpora, the leading contributors included universities of Chinese medicine and their affiliated hospitals, pediatric and maternal–child health institutions, and comprehensive universities. Nanhai Maternity and Children’s Hospital Affiliated to Guangzhou University of Chinese Medicine ranked first in both corpora, while Guangzhou University of Chinese Medicine also appeared among the leading institutions in both datasets. The Chinese-language corpus additionally showed substantial contributions from several institutions located in Henan Province.

### 3.4 Journals

The 10 leading journals in the English-language corpus accounted for 132 publications, representing 57.4% of the 230 records (Table 5). *Chinese Acupuncture & Moxibustion* ranked first with 43 publications (18.7%), followed by the *Journal of Acupuncture and Tuina Science* with 20 publications (8.7%) and the *Chinese Journal of Clinical Rehabilitation* with 18 publications (7.8%). *Acupuncture Research*, the *Chinese Journal of Tissue Engineering Research*, and the *Chinese Journal of Integrated Traditional and Western Medicine* each published nine articles (3.9%). *Neural Regeneration Research* and the *Journal of Traditional Chinese Medicine* each contributed seven publications (3.0%), while the *Chinese Journal of Rehabilitation Medicine* and the *World Journal of Acupuncture–Moxibustion* each contributed five publications (2.2%).

**Table 5:** Top 10 journals by publication output in the English-language corpus of research on traditional Chinese medicine-related interventions for cerebral palsy.

Rank	Journal	Publications	Proportion
1	Chinese Acupuncture & Moxibustion	43	18.7%
2	Journal of Acupuncture and Tuina Science	20	8.7%
3	Chinese Journal of Clinical Rehabilitation	18	7.8%
4	Acupuncture Research	9	3.9%
5	Chinese Journal of Tissue Engineering Research	9	3.9%
6	Chinese Journal of Integrated Traditional and Western Medicine	9	3.9%
7	Neural Regeneration Research	7	3.0%
8	Journal of Traditional Chinese Medicine	7	3.0%
9	Chinese Journal of Rehabilitation Medicine	5	2.2%
10	World Journal of Acupuncture–Moxibustion	5	2.2%

The 10 leading journals in the Chinese-language corpus published 643 articles, accounting for 19.9% of the 3,225 records. *Massage and Rehabilitation Medicine* ranked first with 97 publications (3.0%), followed by the *Journal of Pediatrics of Traditional Chinese Medicine* with 71 (2.2%) and the *Journal of Clinical Acupuncture and Moxibustion* with 70 (2.2%). *Chinese Acupuncture and Moxibustion* and the *Shanghai Journal of Acupuncture and Moxibustion* contributed 69 (2.1%) and 68 (2.1%) publications, respectively. *Chinese Pediatrics of Integrated Traditional and Western Medicine* published 60 articles (1.9%), followed by the *Chinese Journal of Clinical Rehabilitation* with 59 (1.8%), *Guangming Journal of Chinese Medicine* with 53 (1.6%), *Liaoning Journal of Traditional Chinese Medicine* with 49 (1.5%), and the *Chinese Journal of Rehabilitation Theory and Practice* with 47 (1.5%).

The English-language corpus was more concentrated within its leading publication sources, with the top 10 accounting for more than half of all records. In contrast, the top 10 sources in the Chinese-language corpus accounted for one-fifth of the corpus, indicating a broader distribution across publication outlets. In both corpora, the leading sources were primarily related to acupuncture and moxibustion, traditional Chinese medicine, rehabilitation, and pediatric medicine (Table 6).

**Table 6:** Top 10 journals by publication output in the Chinese-language corpus of research on traditional Chinese medicine-related interventions for cerebral palsy.

Rank	Journal	Publications	Proportion
1	Massage and Rehabilitation Medicine	97	3.0%
2	Journal of Pediatrics of Traditional Chinese Medicine	71	2.2%
3	Journal of Clinical Acupuncture and Moxibustion	70	2.2%
4	Chinese Acupuncture & Moxibustion	69	2.1%
5	Shanghai Journal of Acupuncture and Moxibustion	68	2.1%
6	Chinese Pediatrics of Integrated Traditional and Western Medicine	60	1.9%
7	Chinese Journal of Clinical Rehabilitation	59	1.8%
8	Guangming Journal of Chinese Medicine	53	1.6%
9	Liaoning Journal of Traditional Chinese Medicine	49	1.5%
10	Chinese Journal of Rehabilitation Theory and Practice	47	1.5%

### 3.5 Leading authors and collaboration patterns

In the Chinese-language corpus, the most productive authors were Liu Zhenhuan, with 71 publications, followed by Jin Bingxu with 33 and Ma Bingxiang with 32. In the English-language corpus, Liu Z. H. ranked first with 15 publications, followed by Zhao Y. with 12 and Liu Y. with 11. The complete lists of the 10 most productive authors in each corpus are presented in Tables 7, 8.

**Table 7:** Top 10 authors by publication output in the English-language corpus.

Rank	Author	Publications	Proportion, %
1	Liu, Z. H.	15	6.5
2	Zhao, Y.	12	5.2
3	Liu, Y.	11	4.8
4	Li, N.	8	3.5
5	Fu, W. J.	8	3.5
6	Wu, Y.	7	3.0
7	Jin, B. X.	7	3.0
8	Zhang, H. Y.	7	3.0
9	Zhang, Y.	6	2.6
10	Zheng, H.	6	2.6

**Table 8:** Top 10 authors by publication output in the Chinese-language corpus.

Rank	Author	Publications	Proportion, %
1	Liu, Z. H.	71	2.2
2	Jin, B. X.	33	1.0
3	Ma, B. X.	32	1.0
4	Zhao, Y.	30	0.9
5	Wang, X. F.	28	0.9
6	Shang, Q.	26	0.8
7	Huang, M.	25	0.8
8	Gao, J.	24	0.7
9	Shi, B. P.	24	0.7
10	Tai, X. T.	22	0.7

The Chinese-language coauthorship network comprised 5,493 authors and 11,121 coauthorship links. It contained 1,407 connected components, and its largest connected component included 1,775 authors, accounting for 32.3% of the network. The mean degree was 4.05, the average clustering coefficient was 0.671, and 66.2% of publications involved two or more authors. Detailed rankings based on normalized degree centrality, weighted collaboration strength, and largest-component betweenness centrality are provided in Supplementary Table S3.

The English-language coauthorship network comprised 787 authors and 2,809 coauthorship links. It contained 62 connected components, and the largest connected component included 529 authors, representing 67.2% of the network. The mean degree was 7.14, the average clustering coefficient was 0.872, and 93.9% of publications were multi-authored (Table 9).

**Table 9:** Structural characteristics of the coauthorship networks in the Chinese- and English-language corpora.

Metric	Chinese-language corpus	English-language corpus
Number of authors	5,493	787
Number of coauthorship links	11,121	2,809
Network density	0.000737	0.009082
Connected components	1,407	62
Authors in the largest component	1,775	529
Largest-component share	32.3%	67.2%
Mean degree	4.05	7.14
Average clustering coefficient	0.671	0.872
Multi-author publication rate	66.2%	93.9%
Mean authors per publication	2.82	5.00
Isolated authors	706	9

**Figure 2:**
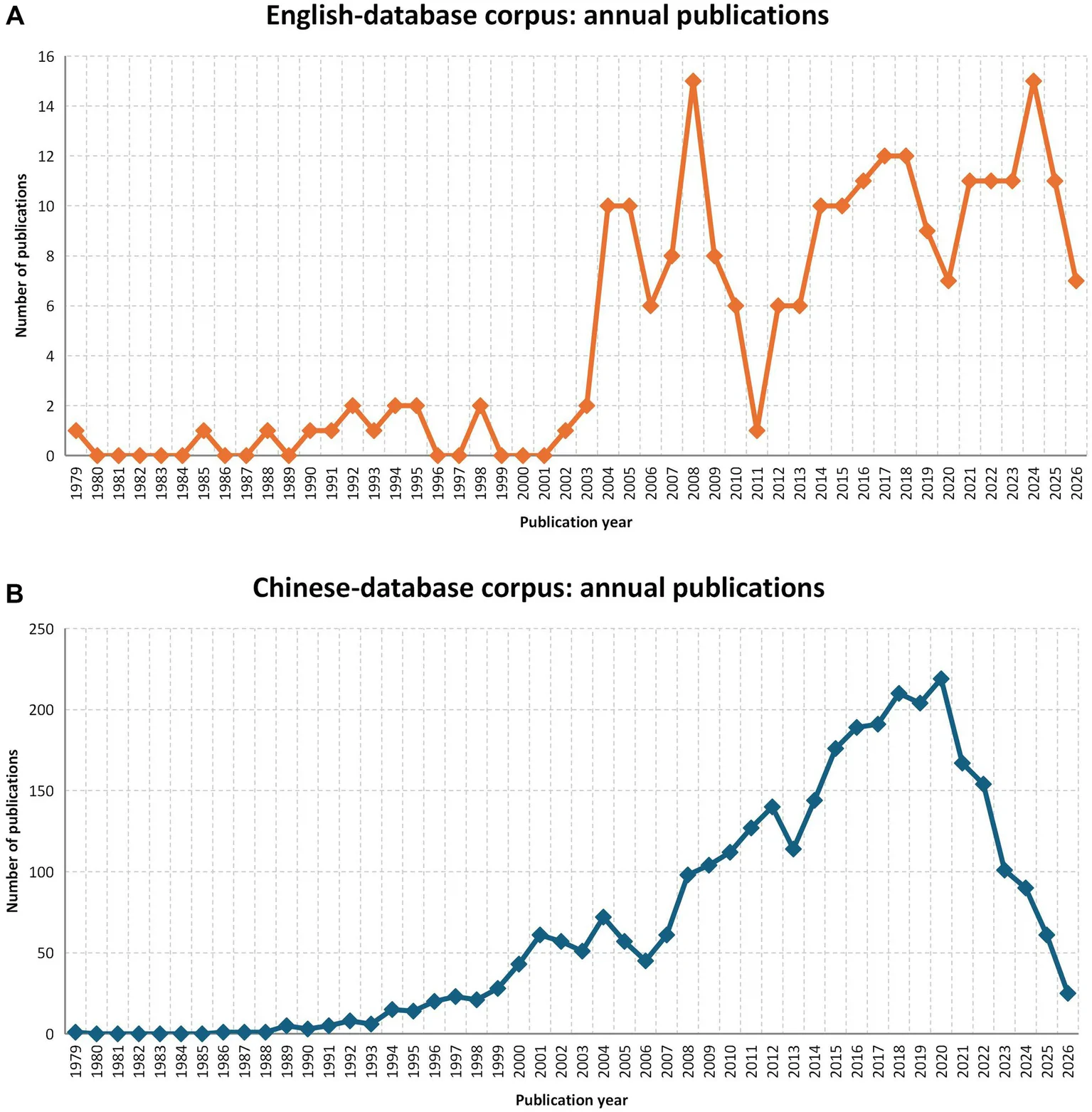
Annual publication trends in English- and Chinese-language research on traditional Chinese medicine-related interventions for cerebral palsy. **(A)** English-language corpus and **(B)** Chinese-language corpus. The complete corpora comprised 230 and 3,225 records, respectively. All records with valid publication-year information were included. Publication counts for 2026 represent an incomplete year and should be interpreted cautiously.

The English-language network therefore showed a larger proportion of authors connected within the principal component, a higher mean number of collaborators per author, and a higher rate of multi-author publications. The Chinese-language network was substantially larger but was divided into a greater number of separate collaboration groups. Publication productivity and structural centrality were not fully aligned, as several authors with moderate publication output occupied relatively prominent bridging positions between collaboration groups (Figure 2).

### 3.6 Keyword co-occurrence and thematic evolution

**Figure 3:**
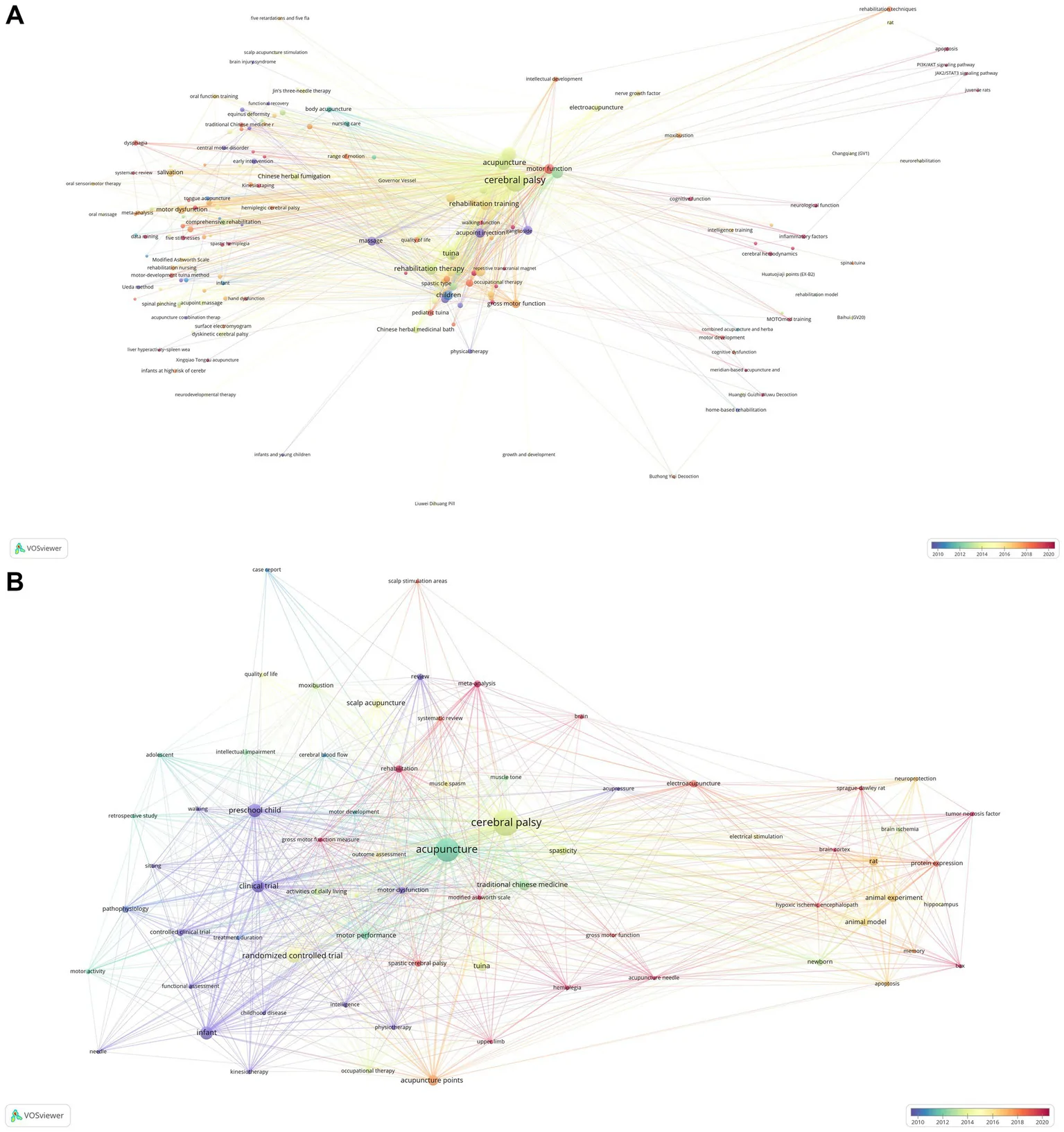
Keyword co-occurrence networks in Chinese- and English-language research on Traditional Chinese medicine-related interventions for Cerebral palsy: **(A)** English-language corpus and **(B)** Chinese-language corpus. In the Chinese-language corpus, 179 of 2,115 standardized keywords met the minimum occurrence threshold of five; in the English-language corpus, 70 of 955 standardized keywords met the same threshold. Node size represents keyword occurrence frequency, links represent keyword co-occurrence relationships, and link thickness indicates co-occurrence strength. Colors indicate clusters identified separately within each corpus. Chinese keyword labels were translated into English after network construction using a predefined one-to-one terminology mapping; this replacement did not alter node identities, occurrence counts, links, link strengths, coordinates, or cluster assignments.

Author keywords and indexed keywords were standardized before analysis by merging synonymous terms and removing non-informative terms using corpus-specific thesaurus files. In the English-language corpus, 70 of 955 standardized keywords met the minimum occurrence threshold of five, whereas 179 of 2,115 standardized keywords met this threshold in the Chinese-language corpus. VOSviewer automatically partitioned the English- and Chinese-language keyword networks into five and 10 clusters, respectively (Figure 3). The thematic labels used below are descriptive summaries rather than labels generated by the software. They were assigned by examining the keywords with the highest occurrence frequencies and total link strengths within each cluster and assessing their semantic coherence.

In the English-language corpus, *cerebral palsy* and *acupuncture* were the most prominent and strongly connected terms. Other frequently occurring or structurally prominent terms included *clinical trial*, *randomized controlled trial*, *preschool child*, *infant*, *traditional Chinese medicine*, *scalp acupuncture*, *tuina*, *motor performance*, and *acupuncture points*. The software-generated clusters represented several related thematic groupings. One grouping concerned clinical evaluation and pediatric motor outcomes, including *clinical trial*, *randomized controlled trial*, *motor performance*, *motor dysfunction*, *activities of daily living*, and the *Gross Motor Function Measure*. A second grouping was centered on acupuncture-related interventions and rehabilitation, represented by *acupuncture*, *scalp acupuncture*, *rehabilitation*, *acupressure*, and *traditional Chinese medicine*. Review-related terms, including *review*, *systematic review*, and *meta-analysis*, formed part of the clinical research and evidence-synthesis structure. Another distinct grouping comprised experimental and mechanistic terms such as *rat*, *animal model*, *animal experiment*, *brain ischemia*, *protein expression*, *tumor necrosis factor*, *apoptosis*, and *neuroprotection*. Overall, the English-language network connected clinical study design, acupuncture-based treatment, motor-function assessment, and experimental mechanism research.

In the Chinese-language corpus, the largest and most strongly connected nodes included *cerebral palsy*, *acupuncture*, *rehabilitation training*, *motor function*, *tuina*, *rehabilitation therapy*, *children*, *muscle tone*, *scalp acupuncture*, and *motor dysfunction*. The cluster structure was more extensive than that of the English-language network and reflected the substantially larger corpus. Major thematic groupings included acupuncture and related techniques, such as *scalp acupuncture*, *electroacupuncture*, *body acupuncture*, *moxibustion*, and *acupoint injection*; tuina and other external TCM therapies, including *pediatric tuina*, *massage*, *acupoint massage*, herbal fumigation, herbal baths, and acupoint application; and rehabilitation and functional assessment, represented by *rehabilitation training*, *rehabilitation therapy*, *motor function*, *gross motor function*, *balance function*, *muscle tone*, and *activities of daily living*. Additional groupings concerned clinical manifestations and cerebral-palsy subtypes, including *spastic cerebral palsy*, *motor dysfunction*, *dysphagia*, *salivation*, and *language disorder*, as well as experimental and physiological research involving *electroacupuncture*, *nerve growth factor*, *cerebral hemodynamics*, inflammatory factors, apoptosis, and intracellular signaling pathways. These patterns indicate that the Chinese-language literature covered a broad combination of clinical TCM interventions, rehabilitation outcomes, symptom-oriented management, and mechanistic investigation.

**Figure 4:**
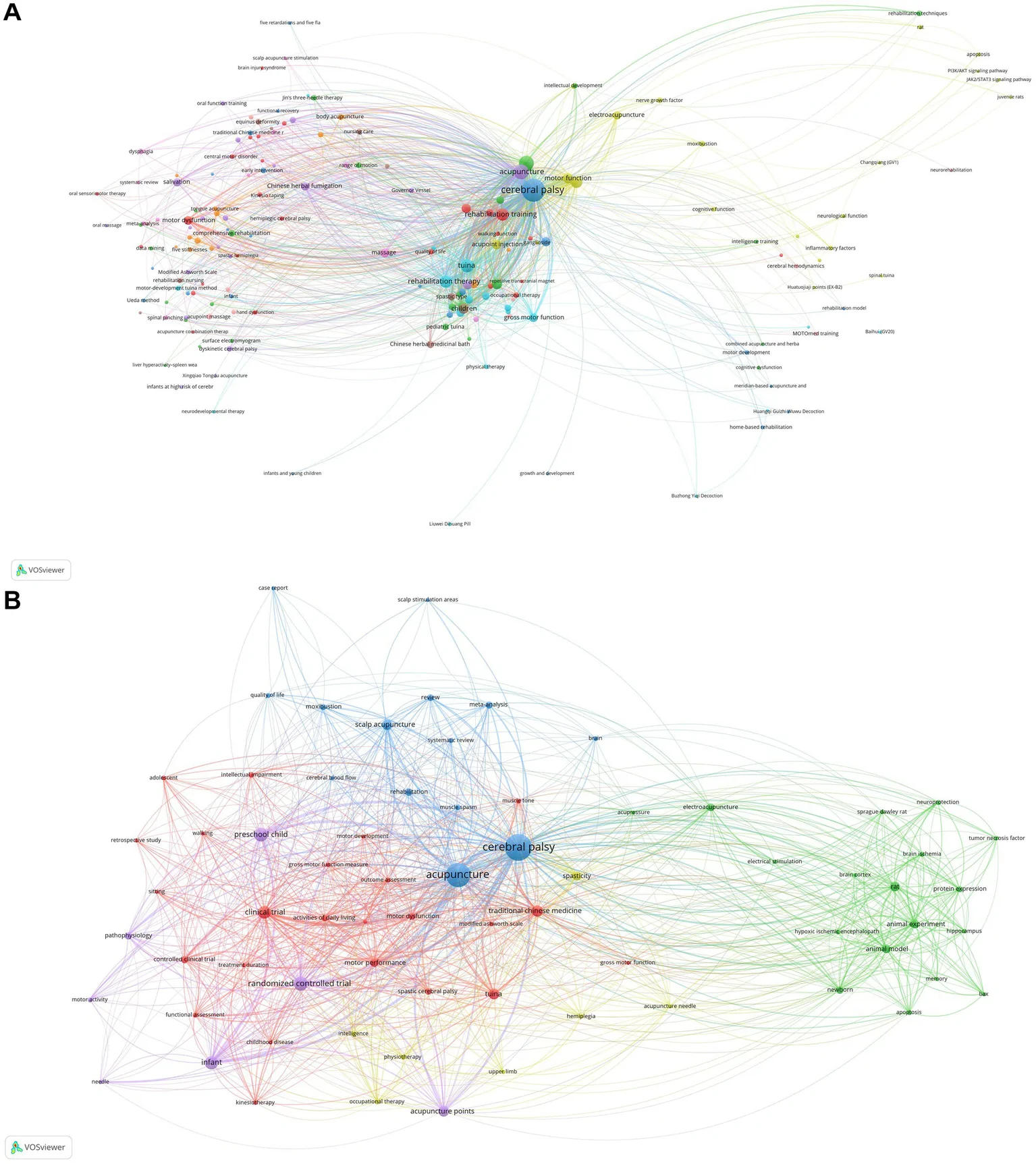
Overlay visualization of keyword evolution in Chinese- and English-language research on traditional Chinese medicine-related interventions for cerebral palsy: **(A)** English-language corpus and **(B)** Chinese-language corpus. The overlay visualizations were generated from the same keyword networks and node sets used in Figure 3. Node size represents keyword occurrence frequency, links represent co-occurrence relationships, and node color indicates the average publication year of documents containing the corresponding keyword. A common color scale from 2010 to 2020 was applied to both panels to facilitate visual comparison of temporal patterns. Chinese keyword labels were translated into English for display purposes without altering the underlying network structure or temporal scores.

The overlay visualizations show the average publication year associated with each keyword and therefore illustrate changes in the temporal distribution of research topics rather than the first appearance of individual terms (Figure 4). In the English-language corpus, terms with relatively earlier average publication years included *infant*, *pathophysiology*, *functional assessment*, *controlled clinical trial*, *motor activity*, and *acupuncture points*. Terms such as *cerebral palsy*, *acupuncture*, *traditional Chinese medicine*, *rehabilitation*, and motor-related outcomes occupied the intermediate period. Keywords with relatively recent average publication years included *systematic review*, *meta-analysis*, *brain*, *electroacupuncture*, *animal model*, *animal experiment*, *protein expression*, *tumor necrosis factor*, *apoptosis*, and *neuroprotection*. This distribution suggests increasing attention to research synthesis and experimental investigation alongside continued clinical evaluation of acupuncture-related interventions.

In the Chinese-language corpus, relatively earlier terms were more frequently associated with general pediatric populations, conventional rehabilitation assessment, and established clinical techniques. The central portion of the temporal map was dominated by acupuncture, tuina, rehabilitation training, cerebral palsy, motor function, muscle tone, and other clinical outcome terms. More recent average publication years were observed for terms related to neurological function, cerebral hemodynamics, inflammatory factors, apoptosis, juvenile animal models, and the PI3K/AKT and JAK2/STAT3 signaling pathways. Several newer rehabilitation concepts, including rehabilitation models, rehabilitation techniques, and technology-assisted training, also appeared toward the recent end of the scale. The Chinese-language literature therefore showed a temporal broadening from predominantly clinical intervention and rehabilitation topics toward more specific functional outcomes, structured rehabilitation approaches, and biological mechanism research.

Taken together, both corpora were centered on acupuncture-related interventions and motor-function rehabilitation, but their thematic structures differed in breadth. The Chinese-language network contained a wider range of specific TCM techniques, external therapies, clinical symptoms, and rehabilitation applications, whereas the English-language network showed a more distinct experimental-model component and a stronger presence of clinical-study and evidence-synthesis terminology. The overlay maps in both corpora indicated increasing attention to standardized functional assessment and mechanistic research. Because the networks were constructed separately, cluster colors and absolute node or link sizes should be interpreted within each corpus rather than compared directly between panels.

### 3.7 Keyword burst analysis

**Figure 5:**
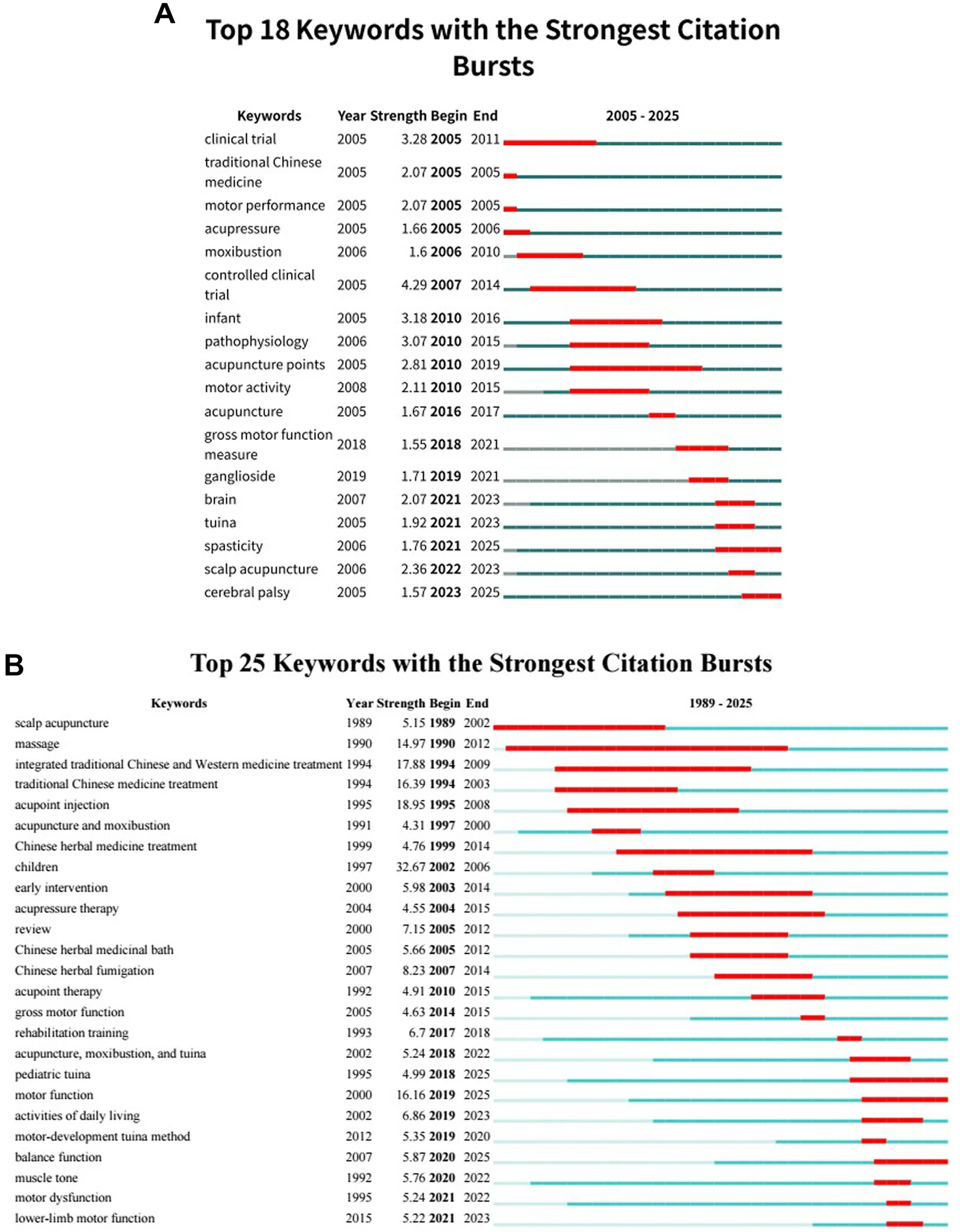
Keyword burst analysis in English- and Chinese-language research on traditional Chinese medicine-related interventions for cerebral palsy. **(A)** The 18 burst keywords detected in the English-language corpus during 2005–2025 and **(B)** The 25 keywords with the strongest bursts in the Chinese-language corpus during 1989–2025. The 2026 records were excluded from burst detection because the publication year was incomplete.

Keyword burst detection was used to identify terms showing marked, time-limited increases in occurrence frequency. The analyses were conducted separately for the Chinese-language corpus over 1989–2025 and the English-language corpus over 2005–2025. Figure 5 presents the 25 strongest burst keywords in the Chinese-language corpus and the 18 burst keywords detected in the English-language corpus.

In the Chinese-language corpus, the early burst pattern was dominated by broad TCM interventions and combined therapeutic approaches. *Scalp acupuncture* showed a prolonged burst from 1989 to 2002, while *massage* remained active from 1990 to 2012. Other prominent early terms included *integrated traditional Chinese and Western medicine treatment* (1994–2009), *traditional Chinese medicine treatment* (1994–2003), *acupoint injection* (1995–2008), *acupuncture and moxibustion* (1997–2000), and *Chinese herbal medicine treatment* (1999–2014). The generic population term *children* showed the highest burst strength (32.67; 2002–2006), followed by *acupoint injection* (18.95), *integrated traditional Chinese and Western medicine treatment* (17.88), and *traditional Chinese medicine treatment* (16.39). These patterns reflect an early concentration on pediatric clinical practice and the application of acupuncture, massage, herbal medicine, and combined treatment modalities.

From the mid-2000s to the mid-2010s, the Chinese-language burst pattern became more diversified. *Early intervention* remained active from 2003 to 2014, while *review*, *Chinese herbal medicinal bath*, *Chinese herbal fumigation*, and *acupoint therapy* emerged between 2005 and 2015. *Gross motor function* showed a short burst during 2014–2015, indicating increasing attention to function-oriented outcome assessment. After 2017, the burst pattern shifted more clearly toward combined rehabilitation approaches and specific functional outcomes. Relevant terms included *rehabilitation training* (2017–2018), *acupuncture and tuina* (2018–2022), *pediatric tuina* (2018–2025), *motor function* (2019–2025), *activities of daily living* (2019–2023), *motor-development tuina method* (2019–2020), *balance function* (2020–2025), *muscle tone* (2020–2022), *motor dysfunction* (2021–2022), and *lower-limb motor function* (2021–2023). Among these recent terms, *motor function* showed the greatest burst strength (16.16), while *pediatric tuina*, *motor function*, and *balance function* remained active through 2025. These findings indicate increasing attention to pediatric tuina and measurable motor, balance, and daily-function outcomes.

In the English-language corpus, early bursts were concentrated on clinical study design, TCM-related interventions, and motor-function assessment. *Clinical trial* showed a sustained burst from 2005 to 2011, while *controlled clinical trial* had the strongest burst in the English-language corpus (strength 4.29; 2007–2014). Other early terms included *traditional Chinese medicine* and *motor performance* in 2005, *acupressure* during 2005–2006, and *moxibustion* during 2006–2010. These terms indicate that the early English-language literature emphasized clinical evaluation of acupuncture-related and other TCM interventions.

Between 2010 and 2019, the English-language burst pattern expanded to include population descriptors, pathophysiological concepts, motor outcomes, and treatment-specific terminology. *Infant* remained active from 2010 to 2016, while *pathophysiology* and *motor activity* continued from 2010 to 2015. *Acupuncture points* showed the longest burst in this phase, extending from 2010 to 2019. This was followed by *acupuncture* during 2016–2017, the *Gross Motor Function Measure* during 2018–2021, and *ganglioside* during 2019–2021.

The most recent English-language bursts included *brain* (2021–2023), *tuina* (2021–2023), *spasticity* (2021–2025), *scalp acupuncture* (2022–2023), and *cerebral palsy* (2023–2025). Among these terms, *spasticity* remained active through 2025 and represented the most sustained recent clinical focus. The recent pattern therefore reflects increased attention to spasticity, tuina, scalp acupuncture, and standardized motor-function assessment. The broad terms *brain* and *cerebral palsy* indicate increased keyword use but provide less specific information about thematic development.

Overall, both corpora showed a temporal transition from broad intervention categories and general clinical evaluation toward more specific treatment modalities and function-oriented outcomes. Recent bursts in the Chinese-language corpus were more strongly associated with pediatric tuina, motor function, balance, and activities of daily living, whereas the English-language corpus showed greater emphasis on spasticity, scalp acupuncture, tuina, and standardized gross-motor assessment. Because the two corpora differed substantially in size and were analyzed using different burst-detection parameters, burst strengths were interpreted within each corpus and were not compared directly.

### 3.8 Parameter sensitivity analyses

Varying the minimum-occurrence threshold affected network size and the visibility of lower-frequency topics but did not alter the five leading keywords in either corpus. In the Chinese-language corpus, increasing the threshold from 3 to 10 reduced the number of retained keywords from 331 to 110, the number of links from 3,342 to 1,728, and total link strength from 13,909 to 11,336. The broad themes related to acupuncture, tuina and other TCM therapies, rehabilitation, functional outcomes, and symptom-oriented management remained identifiable, although lower-frequency techniques and mechanistic subthemes became less visible. In the English-language corpus, the conservative threshold of seven retained 43 keywords, 508 links, and four coherent thematic clusters. The principal themes involving clinical acupuncture research, pediatric and motor-function outcomes, rehabilitation and evidence synthesis, and experimental mechanisms were retained, while peripheral low-frequency terms were reduced.

In the English-language burst analysis, increasing *γ* from 0.5 to 0.6 while retaining a one-year minimum duration reduced the number of detected burst keywords from 18 to 11. The stronger and more sustained early signals, including *clinical trial*, *controlled clinical trial*, *infant*, *pathophysiology*, and *acupuncture points*, were retained. Among the more recent terms, *brain*, *tuina*, and *scalp acupuncture* remained detectable, whereas *spasticity* and *cerebral palsy* were no longer identified. Under the most conservative setting of γ = 0.6 and a minimum duration of 2 years, only *clinical trial*, *controlled clinical trial*, and *infant* remained. These findings indicate that the early, sustained burst signals were comparatively robust, whereas the identification of recent burst terms was sensitive to parameter selection. Accordingly, recent terms detected under the principal setting were interpreted as exploratory temporal signals rather than stable research-frontier indicators.

### 3.9 Integrative thematic synthesis of the research landscape

An author-developed integrative thematic synthesis was conducted to consolidate the findings of the keyword co-occurrence networks, overlay visualizations, and burst analyses. This synthesis was not generated automatically by VOSviewer or CiteSpace and did not constitute an assessment of evidence quality or clinical effectiveness. Five broad research domains were identified: clinical rehabilitation and functional outcomes; spasticity and symptom-oriented management; TCM intervention modalities; clinical research design and evidence synthesis; and experimental and mechanistic research (Table 10).

**Table 10:** Integrative thematic domains in the research landscape of traditional Chinese medicine-related interventions for cerebral palsy.

Research domain	Co-occurrence network support	Overlay visualization support	Burst analysis support	Corpus representation	Integrated interpretation
Clinical rehabilitation and functional outcomes	rehabilitation, rehabilitation training, motor function, gross motor function, Gross Motor Function Measure, balance function, activities of daily living, outcome assessment	Motor-, rehabilitation-, and outcome-related terms occupied intermediate-to-recent positions in both corpora	Chinese: gross motor function, motor function, activities of daily living, balance function, lower-limb motor function; English: Gross Motor Function Measure	Both corpora	Research centered on functional rehabilitation and the assessment of motor, balance, and daily-life outcomes
Spasticity and symptom-oriented management	spastic cerebral palsy, spasticity, muscle tone, modified Ashworth scale, motor dysfunction, dysphagia, salivation	Several symptom-, tone-, and subtype-related terms showed relatively recent average publication years	Chinese: muscle tone, motor dysfunction, lower-limb motor function; English: spasticity	Both corpora	Studies addressed muscle tone, movement impairment, cerebral-palsy subtypes, and specific clinical manifestations
TCM intervention modalities	acupuncture, scalp acupuncture, tuina, pediatric tuina, moxibustion, electroacupuncture, acupoint injection, herbal fumigation and washing	Established interventions occupied earlier and intermediate positions, while several specific techniques remained prominent in recent periods	Chinese: scalp acupuncture, massage, acupuncture and moxibustion, acupoint injection, pediatric tuina, acupuncture and tuina; English: acupressure, moxibustion, acupuncture, tuina, scalp acupuncture	Both corpora; greater modality diversity in the Chinese-language corpus	The literature examined multiple acupuncture-, tuina-, moxibustion-, and externally applied herbal interventions
Clinical research design and evidence synthesis	clinical trial, controlled clinical trial, randomized controlled trial, review, systematic review, meta-analysis	Systematic review and meta-analysis had relatively recent average publication years in the English-language corpus	Chinese: review; English: clinical trial and controlled clinical trial	More explicit in the English-language corpus	The literature included primary clinical evaluation, comparative research designs, and secondary evidence synthesis
Experimental and mechanistic research	animal model, animal experiment, rat, brain ischemia, inflammatory factors, apoptosis, neuroprotection, protein expression, PI3K/AKT, JAK2/STAT3	Multiple animal-, molecular-, and signaling-related terms had relatively recent average publication years	Limited direct burst support; recent English burst for brain	Both corpora; more distinct experimental cluster in the English-language corpus	Experimental studies explored neurological, cellular, inflammatory, and molecular processes associated with TCM-related interventions

Clinical rehabilitation and functional outcomes constituted a central domain in both corpora, as reflected by keywords related to rehabilitation, motor function, gross motor assessment, balance, and activities of daily living. A second domain concerned spasticity and symptom-oriented management, including spastic cerebral palsy, spasticity, muscle tone, motor dysfunction, and related assessment instruments. The third domain encompassed specific TCM intervention modalities, particularly acupuncture, scalp acupuncture, tuina, pediatric tuina, moxibustion, electroacupuncture, and external herbal therapies.

Clinical research design and evidence synthesis formed a further domain, represented by terms such as clinical trial, controlled clinical trial, randomized controlled trial, review, systematic review, and meta-analysis. The final domain involved experimental and mechanistic research, including animal models, brain ischemia, apoptosis, neuroprotection, inflammatory factors, protein expression, and intracellular signaling pathways. Together, these domains summarize the principal thematic structure of the literature rather than indicating a hierarchy of evidence or the relative effectiveness of the included interventions.

## 4 Discussion

### 4.1 Interpretation of the principal patterns

The Chinese- and English-language corpora showed different trajectories of development rather than a single, uniform pattern of growth. Publication activity in the Chinese-language corpus expanded earlier and more rapidly, particularly from the late 2000s onward, and reached its highest level around 2020. By contrast, the English-language literature remained substantially smaller and more variable, although a limited but sustained body of publications became visible after the mid-2000s. These differences are likely to reflect the interaction of clinical practice, research infrastructure, policy support, and publication pathways rather than differences in research interest alone.

China’s predominance should therefore not be attributed solely to the historical origin of TCM. TCM modalities are embedded in a relatively extensive network of Chinese medicine universities, affiliated hospitals, pediatric hospitals, rehabilitation departments, and specialist clinical services, creating more opportunities for routine clinical application, investigator-initiated studies, postgraduate research, and domestic publication. This interpretation is consistent with the institutional results, in which pediatric hospitals and universities or hospitals of Chinese medicine were major contributors. National policy may also have reinforced this research environment. China’s 14th Five-Year Plan for TCM development called for expansion of TCM rehabilitation services, integration of traditional and contemporary rehabilitation technologies, construction of national clinical and research platforms, increased support for TCM research through national science and technology programs, and diversified financial investment (17). These measures provide a plausible institutional context for sustained research activity, although the present study did not analyze funding acknowledgements and therefore cannot quantify the independent effect of funding or policy on publication output.

Database and language structures also contributed to the observed distribution. The inclusion of CNKI, Wanfang, and VIP enabled extensive retrieval of Chinese-language studies that would not necessarily be represented in international multidisciplinary databases. Conversely, publication in English-language journals generally requires international dissemination, English-language reporting, and compliance with journal-specific methodological and reporting expectations. The much larger Chinese-language corpus therefore reflects both the underlying concentration of research activity and the ability of the search strategy to capture domestic publication channels. It should not be interpreted as a direct measure of the global clinical use or comparative acceptance of TCM interventions.

The post-2020 decrease in Chinese-language publication output should also be interpreted cautiously. Bibliometric data alone cannot determine whether it reflects temporary disruptions in clinical research, changes in publication and indexing practices, consolidation of local studies, or a genuine reduction in activity. In addition, the incomplete 2026 publication year creates right-censoring at the end of the series. The observed decline should therefore be described as a publication pattern rather than evidence that interest in the topic has diminished.

The coauthorship results indicate different modes of knowledge production across the two corpora. The Chinese-language network was comparatively fragmented, with numerous components, isolates, and single-author publications, whereas the English-language network showed a higher collaboration rate and a larger proportion of authors connected within its principal component. One possible explanation is that a substantial part of the Chinese-language literature originated from local or single-center clinical practice, while English-language publication more often involved larger multidisciplinary teams and collaboration among clinical, rehabilitation, experimental, and methodological researchers. Nevertheless, the concentration of output within several recurring teams in both corpora indicates that broader multicenter and cross-national networks remain underdeveloped. Author-name ambiguity, particularly initial-based names in the English corpus and unresolved homonyms in the Chinese corpus, also warrants caution when interpreting individual network positions.

The temporal development of research topics can be understood as a process of disciplinary maturation. Early studies were organized around broad intervention categories and routine clinical applications, including acupuncture, massage, herbal therapies, and combined TCM–rehabilitation treatment. As the literature accumulated, attention increasingly shifted toward measurable functional outcomes, including motor function, balance, muscle tone, activities of daily living, and standardized gross-motor assessment. The later visibility of systematic reviews, meta-analyses, animal models, molecular pathways, apoptosis, inflammatory factors, and neuroprotection reflects two parallel demands: synthesis of an expanding clinical literature and greater biological explanation of intervention-related phenomena. This parallel development is consistent with the broader bench-to-clinical-practice orientation described for complementary therapies in neurological disorders (18).

This transition is also consistent with the wider emphasis in rehabilitation research on functioning-related data, robust evidence generation, and multidisciplinary research networks. WHO’s Rehabilitation 2030 initiative identifies research capacity, functioning information, and international partnerships as central components of stronger rehabilitation systems (19). However, the sensitivity analyses showed that recently detected burst terms were less stable than longer-duration historical bursts. Recent burst keywords should therefore be interpreted as topics that received increased attention near the end of the observation period, not as confirmed future research directions.

### 4.2 Theoretical, clinical, research, and policy implications of the thematic framework

The integrative thematic synthesis identified five related domains: clinical rehabilitation and functional outcomes; spasticity and symptom-oriented management; TCM intervention modalities; clinical research design and evidence synthesis; and experimental and mechanistic research. The significance of this framework lies not in establishing an evidence hierarchy, but in showing how the research field is organized across clinical targets, intervention types, knowledge-production methods, and biological explanations. The framework therefore provides a conceptual bridge between the separate keyword networks, overlay patterns, and burst results.

#### 4.2.1 Theoretical implications

From a theoretical perspective, the five-domain structure demonstrates that research on TCM-related interventions for CP cannot be represented adequately as a single intervention category. The domain of TCM intervention modalities describes what is delivered; clinical rehabilitation and spasticity-related domains describe which functional problems are targeted; clinical research design and evidence synthesis describe how knowledge is generated and consolidated; and mechanistic research addresses how observed biological effects are explained. This multidimensional structure is more informative than treating acupuncture, tuina, herbal treatment, and other modalities as interchangeable components of a homogeneous field.

The framework also reveals an incomplete connection between these levels. Clinical studies were predominantly organized around motor and tone-related outcomes, whereas mechanistic studies commonly focused on animal models, inflammatory pathways, apoptosis, neurotrophic factors, or signaling pathways. The two bodies of research were thematically related but were not necessarily connected through shared clinical phenotypes or prespecified translational hypotheses. A more mature research model would connect the intervention protocol, CP subtype, functional target, biological endpoint, and clinically meaningful outcome within the same conceptual pathway.

#### 4.2.2 Clinical implications

The clinical value of the framework is organizational rather than confirmatory. Because this bibliometric study did not assess treatment effects, risk of bias, adverse events, or certainty of evidence, the identified domains cannot establish whether any intervention is effective or appropriate for routine care. They do, however, show that TCM-related interventions have usually been investigated as components of rehabilitation and symptom management rather than as a single alternative to conventional rehabilitation.

The prominence of motor function, muscle tone, balance, and activities of daily living reflects important clinical concerns in CP, but also suggests that the literature may have concentrated more heavily on body function and activity than on participation, quality of life, caregiver burden, communication, individualized goals, and long-term development. International guidance for early intervention in CP emphasizes individualized, task- and context-specific intervention, parental involvement, and functionally meaningful goals (20). Future studies should therefore distinguish changes in impairment-level measures from changes that are meaningful to children and families. Caregiver burden and caregiver quality of life should also be considered, particularly when interventions require frequent attendance, home practice, or prolonged caregiver participation. Research in families of children with CP in China has demonstrated close relationships among care burden, social support, coping, and caregiver quality of life (21).

The intervention-modality domain further emphasizes the need to report individual therapies precisely. Acupuncture, scalp acupuncture, electroacupuncture, tuina, pediatric tuina, moxibustion, acupoint injection, and external herbal therapies differ in theoretical basis, delivery procedures, treatment intensity, practitioner requirements, and potential risks. Combining them under a broad TCM label without sufficient procedural detail reduces reproducibility and limits meaningful comparison. Clinical interpretation therefore requires clear reporting of treatment dose, frequency, duration, practitioner qualifications, co-interventions, adherence, and adverse events.

#### 4.2.3 Research implications

The framework indicates that further progress depends less on generating additional isolated publications than on connecting the existing domains. Clinical studies should incorporate standardized functional outcomes, while mechanistic studies should be nested within clinically interpretable designs wherever feasible. Evidence syntheses should distinguish intervention modalities, CP phenotypes, age groups, functional severity, treatment dose, comparator type, and follow-up duration rather than pooling clinically heterogeneous studies under a broad TCM category.

The differences between the two corpora also suggest complementary research strengths. The Chinese-language literature contained a wider range of specific clinical modalities, symptom-oriented applications, and rehabilitation combinations, whereas the English-language literature showed clearer visibility of trial methodology, evidence synthesis, and experimental mechanisms. Cross-language synthesis could therefore benefit from combining the clinical detail available in Chinese publications with internationally standardized methodology and reporting. This requires bilingual terminology harmonization, more complete English titles and abstracts, persistent identifiers, and accessible protocol and data documentation.

#### 4.2.4 Policy implications

At the policy level, the concentration of publications in China suggests that support for TCM rehabilitation may have contributed to substantial research capacity, but publication quantity alone does not guarantee coordinated or internationally transferable knowledge. The next policy priority should be to strengthen common research infrastructure: multicenter networks, prospective trial registration, standardized intervention manuals, shared outcome definitions, data-quality systems, long-term follow-up platforms, and publication of negative or inconclusive findings.

This direction is consistent with both Chinese policy and global rehabilitation priorities. China’s development plan emphasizes TCM rehabilitation centers, integration of TCM and modern rehabilitation, national research platforms, clinical evaluation, and international clinical-trial infrastructure (17). WHO similarly emphasizes robust rehabilitation evidence, functioning-related data, and partnerships across health systems (19). Funding agencies and institutional decision-makers should therefore evaluate projects not only by publication output, but also by protocol quality, registry compliance, data sharing, stakeholder involvement, multicenter participation, and the production of reusable research resources.

### 4.3 Specific research priorities

The findings support several specific priorities for the next stage of research. These recommendations are based on the combined co-occurrence, overlay, burst, collaboration, and thematic-synthesis results rather than on keyword bursts alone.

#### 4.3.1 Development of a core outcome set

Firstly, a Core Outcome Set (COS) should be developed specifically for clinical studies of TCM-related interventions in children with CP. Its scope and consensus process should follow established minimum standards for COS development, including clear definition of the health condition, population, intervention scope, stakeholder groups, and consensus procedures (22). An international multi-stakeholder COS has already been developed for lower-limb orthopedic surgery in children with CP, demonstrating the feasibility of involving patients, caregivers, clinicians, and researchers in outcome prioritization within this population (23). However, its surgical scope means that it cannot be adopted directly for TCM-related rehabilitation studies.

The COS should determine what outcomes must be reported, while a subsequent measurement-consensus process should determine how and when they should be measured. Candidate domains may include motor function, muscle tone, activity and participation, individualized goal attainment, quality of life, communication, pain, caregiver burden, adverse events, treatment acceptability, and long-term development. Existing CP-related COS initiatives in lower-limb orthopedic surgery demonstrate that multi-stakeholder outcome standardization is feasible, but their scopes are too specific to substitute for a COS addressing TCM-related rehabilitation interventions. COS development should follow minimum methodological standards such as COS-STAD.

#### 4.3.2 Prospective registration and transparent reporting

Secondly, interventional studies should be registered prospectively before recruitment in a recognized registry, such as a WHO ICTRP primary registry—including the Chinese Clinical Trial Registry or the International Traditional Medicine Clinical Trial Registry—or ClinicalTrials.gov. (24) Registration records should provide consistent Chinese and English titles, prespecified primary and secondary outcomes, intervention and comparator details, recruitment targets, follow-up periods, and plans for adverse-event reporting. WHO currently recognizes both ChiCTR and ITMCTR within its primary-registry network.

Registration should be accompanied by accessible protocols, statistical analysis plans, detailed intervention manuals, and timely posting or publication of results. Deviations from the registered protocol should be explicitly documented. These steps would improve transparency and reduce ambiguity arising from selective outcome reporting, duplicated publication, or insufficiently described intervention procedures.

#### 4.3.3 Multicenter collaborative networks

Thirdly, the field should establish multicenter collaborative networks rather than relying predominantly on disconnected single-center projects. The co-design of a global clinical trials network for CP provides a directly relevant model for developing shared research priorities, trial infrastructure, common protocols, and international collaboration in this population (25). A practical model would connect leading pediatric hospitals, TCM universities and hospitals, rehabilitation centers, and international CP research institutions through a common master protocol. The network should use shared eligibility criteria, standardized CP subtype and functional-severity classifications, common case-report forms, centralized or independently verified randomization, blinded outcome assessment where feasible, and harmonized follow-up schedules. Research infrastructure should also accommodate community-based recruitment, decentralized follow-up, and low-resource settings. The SMART-CP trial illustrates how community referral systems and telerehabilitation can be incorporated into a pragmatic CP service and research model to improve access and facilitate continued rehabilitation engagement (26).

Such a network would permit adequately powered studies, prespecified subgroup analyses, external validation across healthcare settings, and more reliable evaluation of treatment heterogeneity. It would also enable smaller institutions to contribute standardized data without independently reproducing the entire trial infrastructure. International partners could strengthen methodology, outcome harmonization, and dissemination, while Chinese centers would contribute substantial clinical experience with the relevant intervention modalities.

#### 4.3.4 Integration of mechanistic and clinical research

Fourthly, mechanistic studies should be linked more directly to clinical questions. Animal and laboratory models may be useful for investigating biological pathways, but mechanistic signals should not be interpreted as clinical benefit. Where feasible, clinical trials should include nested mechanistic substudies with prespecified hypotheses, standardized sample collection, and temporal alignment between biological and functional assessments. Neuroimaging, electrophysiology, biomarkers, and molecular measures should be related to CP phenotype, intervention dose, and clinically meaningful outcomes rather than examined as isolated surrogate endpoints. Research programs should also distinguish mechanisms of the TCM intervention from mechanisms attributable to concurrent rehabilitation, spontaneous development, repeated practice, or nonspecific treatment effects. This is particularly important because many included modalities are delivered as adjuncts to complex rehabilitation programs rather than as isolated interventions. Preclinical studies can generate biologically defined hypotheses for subsequent clinical evaluation. For example, electroacupuncture combined with treadmill exercise has been investigated in a neonatal hypoxia–ischemia rat model in relation to corpus-callosum demyelination and oligodendrogenesis (27). This type of study illustrates a possible mechanism-focused design, but animal findings should not be interpreted as evidence of clinical effectiveness in children with CP.

#### 4.3.5 Responsible use of AI-assisted research

Fifthly, artificial intelligence could support multilingual literature processing, terminology harmonization, automated data-quality checks, movement analysis using video or wearable sensors, and integration of clinical, imaging, and biological data. Where an AI component is prospectively evaluated as part of a clinical intervention or decision-support system, the study protocol and trial report should follow the SPIRIT-AI and CONSORT-AI extensions, respectively (28, 29).

AI should not, however, be presented as an autonomous solution to methodological limitations. Algorithms used for participant selection or functional measurement require external validation across CP subtypes, severity levels, ages, languages, and clinical settings. Their outputs should be compared with validated clinical measures and reviewed by qualified clinicians. Data privacy, representativeness, algorithmic bias, explainability, external validation, accountability, model updating, and continuing human oversight should be addressed prospectively, particularly because children with CP constitute a heterogeneous and potentially vulnerable population (30).

Collectively, these priorities would shift the field from a collection of locally conducted, modality-specific studies toward a coordinated research program characterized by common outcomes, transparent registration, multicenter collaboration, clinically anchored mechanisms, and responsible use of digital methods. Such developments would improve comparability and interpretability of future research without presuming the effectiveness of any individual TCM intervention.

## 5 Conclusion

This bibliometric and scientometric analysis mapped the development, collaboration patterns, thematic structure, and temporal evolution of Chinese- and English-language research on traditional Chinese medicine-related interventions for cerebral palsy. The two corpora showed distinct publication and collaboration patterns, while the integrative thematic synthesis identified five broad research domains: clinical rehabilitation and functional outcomes; spasticity and symptom-oriented management; TCM intervention modalities; clinical research design and evidence synthesis; and experimental and mechanistic research. Temporal analyses indicated a gradual broadening from general clinical applications toward standardized functional assessment, evidence synthesis, and mechanistic investigation. These findings describe the organization and evolution of the research literature and do not establish the clinical efficacy, safety, or certainty of evidence for any intervention. Future research should prioritize prospectively registered multicenter studies, standardized intervention reporting, development of a COS, systematic safety assessment, and clinically anchored mechanistic research. The efficacy and safety of individual TCM-related interventions require confirmation through adequately designed randomized controlled trials and rigorous evidence synthesis.

## 6 Limitations

Several limitations should be considered. First, although seven major Chinese and international databases were searched, bibliographic coverage is not exhaustive, and the findings primarily represent Chinese- and English-language publications available through the included databases. Differences in indexing practices and metadata structure also required the two language corpora to be analyzed separately; therefore, absolute network measures and burst strengths should not be compared directly across corpora. Second, despite manual harmonization, residual inconsistencies in author, institutional, and keyword metadata may remain, and the author-developed thematic synthesis inevitably involved some interpretive judgment. Third, visualization and burst-detection results depend partly on software algorithms and parameter settings. Sensitivity analyses supported the broad thematic structure, although lower-frequency topics and recent burst signals were less stable under more restrictive settings. Finally, bibliometric and scientometric analyses describe publication patterns, collaboration structures, thematic organization, and temporal changes, but they do not assess study-level methodological quality, risk of bias, certainty of evidence, clinical efficacy, or safety.

## Data Availability

The original contributions presented in the study are included in the article/Supplementary material, further inquiries can be directed to the corresponding author/s.
