# Supplementary material for: Mapping traditional Chinese medicine-related complementary interventions for cerebral palsy rehabilitation: a bibliometric and visualization analysis
Source: Front Neurol. 2026 Aug 26;17:1882028. doi: 10.3389/fneur.2026.1882028 (PMC13553241; doi:10.3389/fneur.2026.1882028)
Supplement: Supplementary file 1 [file Data_Sheet_1.DOCX]

label	replace by

human

article

controlled study

child

male

female

major clinical study	clinical trial

Chinese medicine	traditional Chinese medicine

nonhuman

treatment outcome

acupuncture point	acupuncture points

massage	tuina

Integrative & Complementary Medicine

Child, Preschool	preschool child

children

animal tissue

clinical effectiveness

Humans

methodology

Neurosciences & Neurology

clinical article

scalp	scalp acupuncture

therapy

Acupuncture Therapy	acupuncture

comparative study

*Acupuncture Therapy	acupuncture

daily life activity	activities of daily living

priority journal

procedures

scoring system

therapy effect

A

China

intermethod comparison

multimodality cancer therapy

unclassified drug

body position

Pediatrics

statistical analysis

upregulation

medicine

prevalence

protein Bax	BAX

Sprague Dawley rat	Sprague-Dawley rat

statistical significance

*Acupuncture Points	acupuncture points

brain derived neurotrophic factor	brain-derived neurotrophic factor

caspase 3	caspase-3

Chinese drug	traditional Chinese medicine

clinical feature

data analysis software

efficacy

enzyme linked immunosorbent assay

follow up

latent period

randomization

animal

blood

Cerebral Palsy/*therapy	cerebral palsy

data analysis

down regulation

electrostimulation	electrical stimulation

herbaceous agent	traditional Chinese medicine

interleukin 6	interleukin-6

mental deficiency	intellectual impairment

meta analysis	meta-analysis

Myospasm	muscle spasm

newborn hypoxia	neonatal hypoxia

protein bcl 2	BCL-2

R246.4

rank sum test

school child	school-age child

Science & Technology - Other Topics

stimulation

stroke

treatment response

TUNEL assay

activation

Acupuncture Therapy/*methods	acupuncture

Acupuncture-moxibustion Therapy	acupuncture and moxibustion

back

brain blood flow	cerebral blood flow

brain circulation	cerebral blood flow

care

caspase 9	caspase-9

cell count

cells

Cerebral Palsy/complications/*therapy

Cerebral Palsy/physiopathology/*therapy

Chinese medicinal formula	traditional Chinese medicine

clinical observation

clinical research

drug effect

function

groups by age

hand

hospital admission

Meta -analysis	meta-analysis

meta analysis (topic)	meta-analysis

microscope

neuromodulin	GAP-43

Oncology

outcomes research

palsy	cerebral palsy

pediatric patient

Pharmacology & Pharmacy

pressure

protein blood level

Psychiatry

public hospital

quantitative analysis

R244.1

R246.6

randomized controlled trial (topic)	randomized controlled trial

real time polymerase chain reaction

recumbency

research

Research & Experimental Medicine

scale

single blind procedure

Spasm	muscle spasm

spine

Surgery

symptom

systematic review (topic)	systematic review

traditional medicine	traditional Chinese medicine

Tui Na	tuina

*Acupuncture Therapy/instrumentation	acupuncture

*Electric Stimulation Therapy	electrical stimulation

*Electroacupuncture	electroacupuncture

*Language Therapy

*Phytotherapy

*Scalp	scalp acupuncture

*Scalp/physiopathology	scalp acupuncture

*Up-Regulation

2',3' cyclic nucleotide 3' phosphodiesterase	2′,3′-cyclic-nucleotide 3′-phosphodiesterase

4 aminobutyric acid	gamma-aminobutyric acid

Acupoint Sticking Therapy	acupoint application

acupuncture treatment	acupuncture

acupuncture: cerebral palsy (CP)	acupuncture

acupunture	acupuncture

Akt signaling	PI3K/AKT signaling pathway

antimicrobial photodynamic therapy

atherosclerosis

bias

Biochemistry & Molecular Biology

blood collection tube

Cerebral

Cerebral Palsy/*complications

Cerebral Palsy/*drug therapy

Cerebral Palsy/*physiopathology/psychology/*therapy

Cerebral Palsy/physiopathology/*psychology/*therapy

Cerebral Palsy/physiopathology/rehabilitation/*therapy

Cerebral Palsy/psychology/*therapy

cerebrovascular accident

chi square test

ChiCTR2200058879

ChiCTR2200059823

chinese acupuncture	acupuncture

classification

clinical outcome

clinical-trials	clinical trial

comparative effectiveness

confocal microscope

definition

diagnostic accuracy

effect size

Electric stimulation therapy	electrical stimulation

Engineering

epidemiology

Ethics Committee of Nuclear Industry 416 Hospital. Animal subjects: All authors

Evaluation Study

expenditure

expression

false discovery rate

Flow cytometry

fluorescence microscope

functional enrichment analysis

gene ontology

General & Internal Medicine

GMAE Version 1.0.

GraphPad Prism 7.0 software

Gross motor	gross motor function

Health Care Sciences & Services

hospital patient

hypoxia inducible factor 1	hypoxia-inducible factor 1

hypoxia inducible factor 1alpha	hypoxia-inducible factor-1 alpha

Hypoxia-Inducible Factor 1, alpha Subunit/metabolism	hypoxia-inducible factor-1 alpha

incidence

Industry 416 Hospital issued approval KJ2022021. The study was approved by the

Infantile cerebral palsy	cerebral palsy

information retrieval

Integrative Medicine

Intellectual Disability	intellectual impairment

interferon induced GTP binding protein Mx2	interferon-induced GTP-binding protein Mx2

interleukin 10	interleukin-10

interleukin 33	interleukin-33

interleukin 4	interleukin-4

interleukin 8	interleukin-8

Interleukin-6/blood	interleukin-6

jiaosun acupoint	acupuncture points

KEGG

Language Development Disorders/etiology/*therapy	language disability

locomotion	gross motor function

Medical

Mental retardation	intellectual impairment

Meridian acupuncture plus massage	acupuncture plus tuina

NanoDrop 2000

National health insurance research database

NCT03921281

neural regeneration	nerve regeneration

Neurology

neuronal regeneration	nerve regeneration

outcome variable

Overview	review

parent

patient compliance

pediatric

pediatric cerebral palsy	cerebral palsy

Pediatric Massage (Tuina)	tuina

periodicity

phosphatidylinositol 3 kinase	phosphatidylinositol 3-kinase

physician

PI3K-AKT pathway	PI3K/AKT signaling pathway

plant extract

plant medicinal product

Points

polymerase chain reaction system

Preschool	preschool child

protein analysis

protein ZO1	ZO-1 protein

Proteome Discoverer 2.1

quality

R language

R223–01 reverse transcription kit

randomised controlled trials	randomized controlled trial

Randomized Controlled Trials	randomized controlled trial

randomized controlled-trial	randomized controlled trial

Rats	rat

Rats, Sprague-Dawley	Sprague-Dawley rat

receptor type tyrosine protein phosphatase C	receptor-type tyrosine-protein phosphatase C

recovery

Rehabilitation Medicine

reliability

repetative transcranial magnetic stimulation	repetitive transcranial magnetic stimulation

Respiratory Tract Infections/complications/drug therapy/*prevention & control	Respiratory Tract Infections

Review literature	review

RevMan 5.2

risk

risk assessment

safely

sample size

scalp acupuncture Yikang therapy	scalp acupuncture

self

self care	self-care

skull

software

spastic cerebral palsy in children	spastic cerebral palsy

Sport Sciences

SPSS 20.0 (version 20.0)

SPSS software version 25.0

STAT3 protein	STAT3

stress activated protein kinase 1	stress-activated protein kinase 1

symptomatology

system

Systematic reviews	systematic review

The massage for relaxing spastic muscles and stimulating the antagonistic muscles	tuina

therapies

time factor

toll like receptor	toll-like receptor

Traditional

Traditional Chinese medicine (TCM)	traditional Chinese medicine

Tropical Medicine

tuina massage	tuina

TUNEL apoptosis assay kit

UltiMate 3000

V type proton ATPase 16 kDa proteolipid subunit	V-type proton ATPase 16 kDa proteolipid subunit

validation process

warming moxibustion	moxibustion

Xcalibur 2.1.2

yanglingquan acupoint	acupuncture points

zhongyaokangfu	rehabilitation

immunohistochemistry

Western blotting

Western blot

animal cell

nerve cell

injection
