# Supplementary material for: Mapping traditional Chinese medicine-related complementary interventions for cerebral palsy rehabilitation: a bibliometric and visualization analysis
Source: Front Neurol. 2026 Aug 26;17:1882028. doi: 10.3389/fneur.2026.1882028 (PMC13553241; doi:10.3389/fneur.2026.1882028)
Supplement: Supplementary file 2 [file Data_Sheet_2.DOCX]

label	replace by

脑瘫	脑性瘫痪

小儿脑瘫	脑性瘫痪

针刺	针刺疗法

痉挛型脑瘫	脑性瘫痪

康复	康复治疗

针灸疗法	针灸

小儿脑性瘫痪	脑性瘫痪

功能训练	康复训练

脑瘫患儿	脑性瘫痪

中西医结合	中西医结合治疗

痉挛性脑瘫	痉挛型脑性瘫痪

头皮针	头针

中医针灸	针灸

推拿疗法	推拿

流涎症	流涎

中医药疗法	中医药治疗

针灸治疗	针灸

针刺治疗	针刺疗法

痉挛型小儿脑瘫	痉挛型脑性瘫痪

康复疗法	康复治疗

推拿手法	推拿

运动障碍	运动功能障碍

智力低下	智力障碍

推拿按摩	推拿

头针疗法	头针

小儿痉挛型脑瘫	痉挛型脑性瘫痪

推拿治疗	推拿

水针	穴位注射

中医推拿	推拿

中西医结合疗法	中西医结合治疗

按摩疗法	按摩

中医疗法	中医药治疗

综合康复治疗	综合康复

按摩治疗	按摩

头针治疗	头针

语言发育迟缓	语言障碍

中医治疗	中医药治疗

meta分析	Meta分析

穴位注射疗法	穴位注射

外治法	中医外治法

中药浴	中药药浴

言语训练	语言训练

cp	脑性瘫痪

药浴	中药药浴

日常生活活动能力	日常生活能力

bobath疗法	Bobath疗法

平衡能力	平衡功能

肢体运动功能	运动功能

bobath法	Bobath疗法

中医康复治疗	中医康复

儿童脑瘫	脑性瘫痪

上肢功能	上肢运动功能

言语障碍	语言障碍

中医针刺	针刺疗法

生活能力	日常生活能力

粗大运动	粗大运动功能

下肢肌张力	肌张力

肌痉挛	痉挛

脑瘫儿童	脑性瘫痪

痉挛程度	痉挛

不随意运动型脑瘫	不随意运动型脑性瘫痪

语言康复训练	语言训练

肌张力低下	肌张力

循经针灸推拿法	针灸推拿

熏洗	中药熏洗

现代康复疗法	康复治疗

痉挛型脑瘫患儿	痉挛型脑性瘫痪

按摩手法	推拿

粗大运动功能测试量表	粗大运动功能量表

现代康复治疗	康复治疗

刘氏小儿推拿	刘氏小儿推拿法

中药水疗	中药药浴

运动能力	运动功能

研究进展	综述

肢体功能	运动功能

水针疗法	穴位注射

步行能力	步行功能

小儿脑瘫康复	康复治疗

gmfm评分	粗大运动功能量表

随机平行对照研究	随机对照试验

不随意运动型	不随意运动型脑性瘫痪

中医综合疗法	中医药治疗

大脑性瘫痪	脑性瘫痪

中西医	中西医结合治疗

下肢功能	下肢运动功能

中医手法按摩	推拿

功能锻炼	康复训练

水疗法	水疗

现代康复训练	康复训练

中医护理	中医康复护理

脑瘫康复	康复治疗

手法按摩	推拿

小儿脑瘫流涎	流涎

脑性瘫痪患儿	脑性瘫痪

智力水平	智力发育

中药熏蒸疗法	中药熏蒸

adl评分	日常生活能力

熏蒸	中药熏蒸

小儿推拿疗法	小儿推拿

中药疗法	中药治疗

物理治疗	物理疗法

gmfm	粗大运动功能量表

作业治疗	作业疗法

生存质量	生活质量

小儿脑瘫运动功能障碍	运动功能障碍

运动功能训练	康复训练

手足徐动型	不随意运动型脑性瘫痪

弱智	智力障碍

推拿点穴	点穴推拿

儿童脑性瘫痪	脑性瘫痪

中医外治	中医外治法

综合康复疗法	综合康复

adl	日常生活能力

鼠神经生长因子	神经生长因子

痉挛指数	痉挛

痉挛性脑性瘫痪	痉挛型脑性瘫痪

bobath	Bobath疗法

传统针灸	针灸

中医按摩	推拿

小儿脑瘫流涎症	流涎

中药蒸汽浴	中药熏蒸

偏瘫型	偏瘫型脑性瘫痪

穴位注射治疗	穴位注射

脑性麻痹	脑性瘫痪

尖足畸形	尖足

口肌训练	口腔功能训练

穴位注射法	穴位注射

中药外洗	中药熏洗

bobath技术	Bobath疗法

康复运动	康复训练

中药洗浴	中药药浴

电针治疗	电针

普通针刺	针刺疗法

痉挛状态	痉挛

motomed智能运动训练	MOTOmed训练

针刺法	针刺疗法

vojta法	Vojta疗法

综述文献	综述

康复功能训练	康复训练

精细运动	精细运动功能

吞咽功能障碍	吞咽障碍

痉挛型小儿脑性瘫痪	痉挛型脑性瘫痪

中医康复综合疗法	中医康复

偏瘫型脑瘫	偏瘫型脑性瘫痪

智力发育水平	智力发育

脑性瘫痪儿童	脑性瘫痪

电针疗法	电针

综合康复训练	综合康复

jak2/stat3信号通路	JAK2/STAT3信号通路

肌肉痉挛	痉挛

现代康复	康复治疗

炎性因子	炎症因子

指压点穴	点穴疗法

motomed训练	MOTOmed训练

中医康复理疗	中医康复

痉挛性	痉挛

传统康复	康复治疗

中西医结合康复	中西医结合治疗

家庭康复训练	家庭康复

痉挛型脑瘫儿童	痉挛型脑性瘫痪

针炙	针灸

生活自理能力	日常生活能力

粗大运动功能评分	粗大运动功能量表

上肢运动功能障碍	上肢运动功能

dq	发育商

粗大运动功能评估量表	粗大运动功能量表

dq评分	发育商

针刺推拿	针灸推拿

中西医治疗	中西医结合治疗

中药蒸气浴	中药熏蒸

智能发育	智力发育

温针	温针灸

推拿法	推拿

水针治疗	穴位注射

pi3k/akt通路	PI3K/AKT信号通路

神经康复疗法	神经康复

vojta疗法	Vojta疗法

粗大运动功能测试	粗大运动功能量表

不随意运动	不随意运动型脑性瘫痪

中药热敷	中药外敷

bdnf	脑源性神经营养因子

内收肌肌张力	肌张力

粗大运动评定量表	粗大运动功能量表

粗大功能	粗大运动功能

中医针灸治疗	针灸

共济失调型	共济失调型脑性瘫痪

凋亡	细胞凋亡

体穴针刺	体针

体针疗法	体针

改良ashworth量表	改良Ashworth量表

下肢痉挛	痉挛

高压氧治疗	高压氧

外治疗法	中医外治法

白介素-6	白细胞介素-6

常规康复治疗	康复治疗

手足徐动型脑瘫	不随意运动型脑性瘫痪

小儿痉挛型脑性瘫痪	痉挛型脑性瘫痪

中医综合康复护理	中医康复护理

护理干预	康复护理

婴幼儿脑瘫	脑性瘫痪

格赛尔发展量表	Gesell发育量表

生命质量	生活质量

pi3k/akt信号通路	PI3K/AKT信号通路

针刺推拿治疗	针灸推拿

语言功能障碍	语言障碍

中西医疗法	中西医结合治疗

nse	神经元特异性烯醇化酶

gesell发育量表	Gesell发育量表

点穴	点穴疗法

吞咽协调训练	吞咽训练

生活活动能力	日常生活能力

小儿脑瘫痪	脑性瘫痪

埋线	穴位埋线

文献综述	综述

gesell量表	Gesell发育量表

中药酊剂蜡疗	中药蜡疗

精神发育迟滞	智力障碍

小儿智力低下	智力障碍

中西医综合治疗	中西医结合治疗

改良ashworth	改良Ashworth量表

中药浴牵拉	中药药浴

肢体运动能力	运动功能

痉摩型	痉挛型脑性瘫痪

经点穴	点穴疗法

神经康复治疗法	神经康复

外敷	中药外敷

智力障	智力障碍

功能改善	运动功能

小儿脑性瘫痪(cp)	脑性瘫痪

儿童言语训练	语言训练

小儿按摩	小儿推拿

小儿流涎症	流涎

儿童步行功能	步行功能

格塞尔婴幼儿发展量表评分	Gesell发育量表

@粗大运动功能	粗大运动功能

痉挛性双瘫型脑瘫	痉挛型双瘫

痉挛性脑瘫儿	痉挛型脑性瘫痪

脑性瘫痪(脑瘫)	脑性瘫痪

粗大运动能力	粗大运动功能

头穴针刺法	头针

单中心单盲随机对照研究	随机对照试验

现代综合康复疗法	综合康复

小儿脑原性瘫痪	脑性瘫痪

中药浴式水疗	中药药浴

活动能力	运动功能

鲍巴斯疗法	Bobath疗法

石蜡复合中药	中药蜡疗

痉挛肌群	痉挛

早期中医康复护理	中医康复护理

炎症反应	炎症因子

中医针刺疗法	针刺疗法

临床进展	综述

改良穴位埋线	穴位埋线

薰洗	中药熏洗

bobath康复疗法	Bobath疗法

中医特色疗法	中医药治疗

偏侧痉挛型脑性瘫痪	偏瘫型脑性瘫痪

bobath神经发育疗法	Bobath疗法

系统评价再评价	系统评价

头针疗法/治疗	头针

盖瑟尔发育评分	Gesell发育量表

jak2/stat3	JAK2/STAT3信号通路

智能低下	智力障碍

随机对照临床试验	随机对照试验

独立生活能力	日常生活能力

痉挛型脑瘫儿	痉挛型脑性瘫痪

下肢功能障碍	下肢运动功能

传统针刺	针刺疗法

脑瘫流涎	流涎

关节被动活动度	关节活动度

体针治疗	体针

gmfm-88评分	粗大运动功能量表

痉挛型双瘫儿	痉挛型双瘫

按摩推拿	推拿

vojta训练	Vojta疗法

学龄期儿童	学龄期

构音训练	语言训练

gmfm量表评分	粗大运动功能量表

ashworth分级法	改良Ashworth量表

motomed虚拟情景训练	MOTOmed训练

居家康复	家庭康复

中药熏蒸治疗	中药熏蒸

痉挛型脑瘫尖足	尖足

常规康复训练	康复训练

小儿混合型脑瘫	混合型脑性瘫痪

炎性细胞因子	炎症因子

双瘫	痉挛型双瘫

gesell量表评分	Gesell发育量表

下肢痉挛改善情况	痉挛

家庭康复护理	康复护理

bobath手法	Bobath疗法

神经生理(bobath)	Bobath疗法

gmfm-66	粗大运动功能量表

蒸汽浴	中药熏蒸

临床护理	康复护理

精细动作	精细运动功能

acupuncture	针刺疗法

高压氧疗法	高压氧

儿童语言障碍	语言障碍

肌肉痉挛程度	痉挛

gesll评分	Gesell发育量表

传统中医治疗	中医药治疗

神经元凋亡	细胞凋亡

重症痉挛型脑瘫	痉挛型脑性瘫痪

粗大运动功能评定量表	粗大运动功能量表

熏洗疗法	中药熏洗

随机对照研究	随机对照试验

儿童不随意运动型脑瘫	不随意运动型脑性瘫痪

中药蜡敷	中药蜡疗

温针灸疗法	温针灸

精细运动能力	精细运动功能

早期康复训练	康复训练

中医综合干预	中医药治疗

徐动型	不随意运动型脑性瘫痪

gmfm量表	粗大运动功能量表

日常活动能力	日常生活能力

口肌康复训练	口腔功能训练

康复训练护理	康复护理

按摩点穴	点穴推拿

小儿脑瘫性运动功能障碍	运动功能障碍

随机对照试验[文献类型]	随机对照试验

tnf-α	肿瘤坏死因子-α

炎症细胞因子与受体	炎症因子

不随意运动型小儿脑瘫	不随意运动型脑性瘫痪

挛性脑瘫患儿	痉挛型脑性瘫痪

peabody精细运动发育量表	Peabody精细运动发育量表

粗大运动功能测试量表88项	粗大运动功能量表

步行痉挛型脑瘫	痉挛型脑性瘫痪

偏瘫型小儿脑瘫	偏瘫型脑性瘫痪

针刺推拿疗法	针灸推拿

下肢肌张力障碍	肌张力

痉挛肌肌张力	肌张力

神经康复训练	神经康复

脑瘫流涎症	流涎

肌张力增高	肌张力

脑瘫患者	脑性瘫痪

ashworth量表评分	改良Ashworth量表

针利治疗	针刺疗法

康复与护理	康复护理

头部运动区	头针

肢体运动功能障碍	运动功能障碍

voita手法	Vojta疗法

头皮针针刺	头针

中医手法	推拿

中药局部熏蒸及药浴	中药熏蒸

小儿脑性瘫痪性尖足	尖足

babath疗法	Bobath疗法

痉挛脑瘫	痉挛型脑性瘫痪

纯西医和中西医结合治疗	中西医结合治疗

中医药外治疗法	中医外治法

改良ashworth痉挛	改良Ashworth量表

改良ashworth(mas)量表	改良Ashworth量表

头针/治疗应用	头针

单侧痉挛型脑瘫	偏瘫型脑性瘫痪

gmfm66	粗大运动功能量表

针剌疗法	针刺疗法

穴位封闭疗法	穴位注射

小儿智能发展商数	发育商

物理康复训练	康复训练

电针刺激	电针

opt口部肌肉训练	口腔功能训练

头部针炙	头针

水针注射	穴位注射

总发育商	发育商

康复锻炼	康复训练

护理效果	护理

按摩方法	推拿

头部针刺疗法	头针

脑性瘫痪痉挛型双瘫	痉挛型双瘫

综合发育商	发育商

pi3k/akt	PI3K/AKT信号通路

艾条灸	艾灸

gmfm粗大运动功能测试量表	粗大运动功能量表

头皮针刺	头针

下肢站走能力	步行功能

体穴针刺法	体针

医学按摩	推拿

肌张力低下型脑瘫	肌张力低下型脑性瘫痪

点穴按摩	点穴推拿

gmfm-88量表评分	粗大运动功能量表

粗大运动功能测试量表(gmfm)	粗大运动功能量表

小儿步行功能	步行功能

中医穴位按摩	穴位按摩

脑瘫儿	脑性瘫痪

循经针刺推拿法	针灸推拿

痉挛型脑瘫痪	痉挛型脑性瘫痪

小儿运动功能障碍	运动功能障碍

康复护理训练	康复护理

关节活动范围	关节活动度

穴位点刺	点穴疗法

肢体活动能力	运动功能

熏蒸治疗	中药熏蒸

头部针刺	头针

肢体痉挛	痉挛

痉挛型脑性瘫痪患儿	痉挛型脑性瘫痪

fmfm评分	精细运动功能量表

关节活动	关节活动度

生活自理	日常生活能力

脑性瘫疾	脑性瘫痪

肌张力过低	肌张力

点穴治疗	点穴疗法

脑性瘫痰	脑性瘫痪

独立步行能力	步行功能

智力障碍呆证	智力障碍

神经康复技术	神经康复

穴位按摩治疗	穴位按摩

ashworth评分	改良Ashworth量表

中西医综合康复治疗	中西医结合治疗

早期综合康复训练	综合康复

学龄前儿童	学龄前

中西医结合治疗法	中西医结合治疗

改良ashworth痉挛评定量表	改良Ashworth量表

中草药	中药治疗

中医穴位埋线	穴位埋线

穴位点按	点穴疗法

中医综合护理	中医康复护理

中度痉挛型脑瘫	痉挛型脑性瘫痪

德国motomed	MOTOmed训练

脑性瘫痪不自主运动型	不随意运动型脑性瘫痪

口部肌肉训练	口腔功能训练

功能康复训练	康复训练

穴位封闭	穴位注射

医院–家庭康复模式	医院-家庭康复模式

针炙疗法	针灸

改良ashworth痉挛量表(mas)	改良Ashworth量表

推拿按摩疗法	推拿

ngf	神经生长因子

婴幼儿和儿童期	婴幼儿

肌群肌张力	肌张力

全面康复	综合康复

双瘫型小儿脑性瘫痪	痉挛型双瘫

jak/stat通路	JAK2/STAT3信号通路

cerebral palsy	脑性瘫痪

格赛尔发育量表	Gesell发育量表

小儿脑瘴	脑性瘫痪

bobath:疗法	Bobath疗法

中药浴水疗	中药药浴

手精细运动	精细运动功能

推拿电针治疗	针灸推拿

中医推拿按摩	推拿

小儿脑痪	脑性瘫痪

(gmfm)	粗大运动功能量表

传统针刺法	针刺疗法

传统康复疗法	康复治疗

il-6	白细胞介素-6

vojta神经发育疗法	Vojta疗法

康复运动疗法	康复训练

小儿脑瘫患者	脑性瘫痪

痉挛分级	痉挛

头针针刺	头针

白细胞介素6	白细胞介素-6

motomed智能训练系统	MOTOmed训练

脑瘫失语	语言障碍

温针疗法	温针灸

吞咽功能训练	吞咽训练

传统康复应用	康复治疗

中医手法治疗	推拿

88项粗大运动功能量表	粗大运动功能量表

头针运动区	头针

点穴推拿治疗	点穴推拿

语言发展障碍	语言障碍

刘氏推拿	刘氏小儿推拿法

小儿脑性瘫瘫	脑性瘫痪

中西医结合系统治疗	中西医结合治疗

穴注	穴位注射

中药熏洗疗法	中药熏洗

小腿三头肌肌张力	肌张力

中药水浴	中药药浴

神经生长因子蛋白表达	神经生长因子

蒸气浴	中药熏蒸

中医特色康复干预	中医康复

中医综合康复	中医康复

改善关节活动度	关节活动度

srj口肌训练	口腔功能训练

婴幼儿发育商评分	发育商

精细运动功能评估量表(fmfm)	精细运动功能量表

1岁以内	婴儿

中西医结合护理干预	中医康复护理

整合康复	综合康复

康复运动训练	康复训练

婴幼儿期	婴幼儿

徐动倾向	不随意运动型脑性瘫痪

日常生活自理能力	日常生活能力

中药药浴(水疗热浴)	中药药浴

修订ashwotrh量表	改良Ashworth量表

@鼠神经生长因子	神经生长因子

精神发育迟缓	智力障碍

按摩结合针灸	针灸推拿

中医综合康复疗法	中医康复

痉挛型患儿	痉挛型脑性瘫痪

下肢运动功能障碍	下肢运动功能

精细运动功能评估量表	精细运动功能量表

针刺法治疗	针刺疗法

经穴位按摩法	穴位按摩

修订ashworth痉挛评定表	改良Ashworth量表

电体针	电针

经穴推拿	推拿

儿童

治疗

疗效

临床疗效

临床观察

临床效果

疗效观察

综合治疗

中医

患儿

效果

穴

儿科

治疗效果

脑性瘫痪/针灸疗法

综合疗法

临床研究

治疗方法

名医经验

康复效果

应用效果

瘫痪

应用

穴位

治疗结果

脑性瘫痪/中医药疗法

预后

中医药

手法

脑性瘫痪/康复

现代康复理念

斜视

诊断

柳少逸

临床应用

治疗后

现代医学

运动

听力障碍

上肢

影响

选穴规律

脑性

早期

传统医学

临床经验

脑性瘫痪/治疗

效果分析

焦虑

干预

有效率

药物治疗

对照治疗观察

中风

药物疗法

干预效果

文献研究

病因

实验动物

三维步态分析

发育

分析

辅助治疗

常见

王国才

中医理论

痉挛型脑瘫/中医药疗法

头部

脑性瘫痪/中西医结合疗法

聚类分析

脑

影响因素

评估

脑瘫/针灸疗法

治疗应用

同步治疗

疗法

临床资料

脑性瘫痪/按摩疗法

临床

康复疗效

语言障碍/针灸疗法

临床价值

脑性瘫痪/针刺疗法

听觉障碍

早期诊断

作用

取穴

脑性瘫痪/穴位疗法

有效性

价值

年龄

早期发现

围产期

临床分析

发育水平

治疗有效率

中医学

康复医院

儿科学

小儿脑瘫/治疗

中风后遗症

儿

脑性瘫痪/药物疗法

注射

临床有效性

临床症状

对比分析

应用价值

体征

观察

家庭

治疗总有效率

医生

康复医疗

效果观察

头颈部

康复应用

马若飞

概况

儿重

总有效率

痉挛型脑瘫/中西医结合疗法

新生儿肺炎

国家中医药管理局

秦敏

疗效分析

结论

常规治疗

观察组

抑郁症

主要表现

出生后

东莨菪碱/治疗应用

意向处理分析

新生

障碍

耳廓

治疗小儿

研究

改善效果

联合治疗

综合性治疗

患者

游戏设计

疗效评价

早期症状

康复医学

综合征

刘振寰

儿子

位点注射

脑性瘫痪/并发症

功能障碍

满意度

临床表现

家长

康复作用

抑郁

理论研究

洪学滨

脑型瘫痪/中西医结合疗法

医学

病因病机

脑血管意外

新型冠状病毒肺炎

性瘫痪

中国传统/历史

急性肠炎

脑瘫/穴位疗法

意义

阳经取穴

广州中医药大学第一附属医院

精制脑组织液/治疗应用

优秀论文证书

@amie方案

外科学

黄宗勖

贫困地区

病毒

时间

麻痹

针刺/治疗应用

文献

第一附属医院

学术研讨会

发育理论

江苏省无锡市

价值研究

中国

早期诊治

广州市

简称

规律

方法

小儿患者

神经生长因子/治疗应用

病因学

中药药浴/护理

传统方法治疗

针具

男性患儿

治疗规律

理论依据

小儿脑瘫/康复

康复治疗效果

治疗特点

大医院

显效率

可行性研究

对症取穴

结合应用

先天感应神经性耳聋

项目

对比观察

手

疗效评定标准

治疗前

脑卒中

儿科疾病

理论探析

骨密度/针灸效应

孩子

上呼吸道感染

相关性

药物

癫痫

病程

活动

防治

神经干细胞/病理学

复方丹参片/治疗应用

祛风药/治疗应用

功能区

市级医院

脑复素/治疗应用

快速达到康复

配合

合作

生长相关蛋白/针灸效应

脑瘫/针刺疗法

案例背景

平顶山地区

优势分析

序贯分析

综合措施

反复呼吸道感染

胃肠道感染

步态/针灸效应

区域

心脑血管病

三联疗法

经验总结

临床总结

丁鸿山

评定量表

治疗效果分析

发育情况

日常生活活动能力/针灸效应

红外线照射/护理

青州市

黄修武

社区康复

传统中医理论

运动障碍/穴位疗法

监护人

临床实践

评价量表

合并

半身不遂

儿科常见病

功能评价

康复医学科

探究

脊髓

特效疗法

实践分析

健康教育

康复法治疗

游戏

护士

临床过程

泄泻/针灸疗法

康复中心

症状和体征

中医研究院

穴位配伍

肠应激综合征

低

类型

特色治疗

汗证

诊断标准

脑卒中后遗症/中医药疗法

对照观察

体会

医院康复

针刺疗效

康复法

取穴机理

病机

世界

@剪刀步

治疗体会

小儿抽动症

病例分析

三结合康复模式

治疗师

常规护理

脑型瘫痪/运动疗法

脑性麻痹／穴位疗法

颈椎病

注册登记研究

单一被试

农村

李家康

加强护理

显著性

作用探讨

特发性便秘

@柔肝舒筋汤

张振宇

陕西省

水平

治疗观察

@神经干细胞移植

回顾性临床分析

机制比较研究

吸入性肺炎

推拿经验

流涎/并发症

刺激点

/针灸疗法

的临床观察

疗效显著

儿童病

祖国

脑性瘫痪/中药疗法

促进

小儿脑瘫中

2009年

护理满意度

取穴配伍

焦氏头针治疗法

脊柱

外伤性截瘫

吴旭

病历回顾性研究

临床治疗有效率

个性化康复治疗

临床作用

治疗特征

疗效报告

精神分裂症

治疗前后

基础治疗

呼吸道反复感染

皮质盲/针灸疗法

@痉瘫熏蒸剂

机构

马达加斯加

卫生宣教

王肇普

训练效果

改善

中医经络理论

传统疗法

幼儿教育

脑瘫/药物

@上田正疗法

疗程

文献质量评价

人身

临床文献

多学科研究

呼吸道感染

无视力

流涎/针刺疗法

不同年龄段

张卫东

王雪峰

发育时期

小儿腹泻

影响效果

家庭教育

修复

小儿脑瘫/中医药疗法

评估结果

临床意义

关节炎

上升

教育

例分析

医学康复

女儿

心脑血管急症

操作法

脑/中药疗法

临床运用

理论基础

运动区

临床对比观察

诊断方法

头颅

对比疗效

@上田疗法/治疗应用

经验

脑性瘫痪/护理

统计分析

当今社会

护理观察

经穴

活血药/治疗应用

纳络酮/治疗应用

学龄期教育

展望

护理体会

癫痫发作

小儿癫痫

放弃治疗

综合方法

脑瘫/中医药疗法

卒中

经验规律

中医院

人类

张家维

于海波

儿童患者

主要症状

治疗中心

科学依据

儿童群体

中国传统

动物

综合

中国中医研究院

宫长祥

痕量元素/针灸效应

柳少逸经验

脑活素/治疗应用

医学会

特定部位

骨科

失眠

临床治疗效果

流涎/中医药疗法

山东省

呃逆

综合运用

冠军

炎性疾病

下肢

因子分析

病因分析

超早期

干预手段

不同部位

有效治疗

福建省漳州市

@熏蒸机/治疗应用

应用位点

河南中医学院

主穴

脑性瘫痪／针灸疗法

围生期

脑型瘫痪/按摩疗法

运动障碍/针灸疗法

小儿急性偏瘫

脑性瘫痪/中西医结合

■屈肌

应用进展

突触素/针灸效应

脑性瘫痪/诊断

互动游戏

治疗小儿脑瘫

早期教育

视神经萎缩

妇幼保健

脑瘫语言障碍/治疗

客观

王雪峰教授

@王雪峰

功能

应用概况

小儿疾病

特长

医学中国传统

类风湿/针灸疗法

患儿家属

后脑部

步态分析

规范化治疗

性疾病

视力障碍

动作

小儿脑瘫/综合疗法

模型

个案研究

病人

急性细菌性痢疾

针灸培训班

小儿姿势与运动评价

临床评估

胃食管返流病

脑性瘫痪/中药疗法/护理

支气管炎

生长发育期

诱发电位/针灸效应

名家经验

王民集

临床应用效果

小儿泄泻

李学耕

李平

例临床观察

焦顺发

疗效比较

张仲景

治疗原则

脑瘫/并发症

大脑性

治疗疗效

治疗手段

日常生活护理

应用分析

机制研究

杨骏

案例

对症支持治疗

肠道疾病

反复感染

小儿脑瘫/针炙法

标准化

出生前

专业委员会

脑性瘫痪/推拿疗法

国权

实施效果

体会报告

对照组

发育状况

注射治疗

疗效对比

脑/中西医结合疗法

学习

报告质量评价

临床体会
