# Supplementary material for: Mapping traditional Chinese medicine-related complementary interventions for cerebral palsy rehabilitation: a bibliometric and visualization analysis
Source: Front Neurol. 2026 Aug 26;17:1882028. doi: 10.3389/fneur.2026.1882028 (PMC13553241; doi:10.3389/fneur.2026.1882028)
Supplement: Supplementary file 3 [file Table_1.DOCX]

WOS=167 (from inception to July 20, 2026)

| **Search set** | **Query** |
| --- | --- |
| #1 | TS=("cerebral palsy" OR "cerebral palsies" OR "spastic diplegia" OR "spastic hemiplegia" OR "dyskinetic cerebral palsy" OR "athetoid cerebral palsy" OR "athetotic cerebral palsy" OR "mixed cerebral palsy") |
| #2 | TS=( "traditional Chinese medicine" OR "Chinese traditional medicine" OR TCM OR "Chinese medicine" OR "integrated Chinese and Western medicine" OR "integrated traditional Chinese and Western medicine" OR "Chinese herbal medicine*" OR "Chinese herbal drug*" OR "Chinese medicinal herb*" OR "Chinese herbal formula*" OR "Chinese herbal prescription*" OR "Chinese herbal decoction*" OR "traditional Chinese herbal medicine" OR "Chinese patent medicine*" OR "Chinese proprietary medicine*" OR "Chinese phytotherap*" OR "Chinese materia medica" OR acupunct* OR electroacupunct* OR "scalp acupuncture" OR "head acupuncture" OR "auricular acupuncture" OR "ear acupuncture" OR "warm needl*" OR "fire needl*" OR moxibust* OR "warm moxibustion" OR "suspended moxibustion" OR tuina OR "Tui Na" OR "pediatric tuina" OR "Chinese massage" OR "traditional Chinese massage" OR cupping OR "cupping therapy" OR "wet cupping" OR "dry cupping" OR "fire cupping" OR "gua sha" OR guasha OR "scraping therapy" OR ( acupoint* NEAR/3 ( application OR "application therapy" OR sticking OR plaster* OR patch* OR "herbal patch*" OR cataplasm* OR poultice* ) ) OR "acupoint application therapy" OR "acupoint sticking therapy" OR "acupoint herbal application" ) |
| #3 | #1 AND #2 |

Scopus=448 (from inception to July 20, 2026)

( TITLE-ABS-KEY ( "cerebral palsy" OR "cerebral palsies" OR "spastic diplegia" OR "spastic hemiplegia" OR "dyskinetic cerebral palsy" OR "athetoid cerebral palsy" OR "athetotic cerebral palsy" OR "mixed cerebral palsy" ) )

AND ( TITLE-ABS-KEY ( "traditional Chinese medicine" OR TCM OR "Chinese medicine" OR ( ( "Chinese herbal" OR "Chinese herb*" OR herb* ) W/3 ( medicine OR therap* OR formula* OR prescription* OR decoction* ) ) OR "Chinese patent medicine" OR "Chinese proprietary medicine" OR phytotherap* OR "materia medica" OR ( acupunct* OR electroacupunct* OR "scalp acupuncture" OR "auricular acupuncture" OR "warm needl*" OR "fire needl*" ) OR ( moxibustion OR "warm moxibustion" OR "suspended moxibustion" ) OR ( tuina OR "Chinese massage" OR "pediatric tuina" OR "Tui Na" ) OR ( cupping OR "wet cupping" OR "dry cupping" ) OR ( gua sha OR "scraping therapy" ) OR ( acupoint* W/3 ( application OR plaster OR patch OR "herbal patch" OR cataplasm* OR poultice* ) ) ) )

Pubmed=212 (from inception to July 20, 2026)

#1

(

"Cerebral Palsy"[Mesh]

OR "cerebral palsy"[tiab]

OR "cerebral palsies"[tiab]

OR "spastic diplegia"[tiab]

OR "spastic hemiplegia"[tiab]

OR "dyskinetic cerebral palsy"[tiab]

OR "athetoid cerebral palsy"[tiab]

OR "athetotic cerebral palsy"[tiab]

OR "mixed cerebral palsy"[tiab] )

#2

(
"Medicine, Chinese Traditional"[Mesh]
OR "Drugs, Chinese Herbal"[Mesh]
OR "Acupuncture Therapy"[Mesh]
OR "Electroacupuncture"[Mesh]
OR "Acupuncture, Ear"[Mesh]
OR "Moxibustion"[Mesh]
OR "Cupping Therapy"[Mesh]
OR "Phytotherapy"[Mesh]
OR "traditional Chinese medicine"[tiab]
OR "Chinese traditional medicine"[tiab]
OR "Chinese medicine"[tiab]
OR TCM[tiab]
OR "integrated Chinese and Western medicine"[tiab]
OR "integrated traditional Chinese and Western medicine"[tiab]
OR "integrative Chinese medicine"[tiab]
OR "Chinese herbal medicine*"[tiab]
OR "Chinese herbal drug*"[tiab]
OR "Chinese medicinal herb*"[tiab]
OR "Chinese herbal formula*"[tiab]
OR "Chinese herbal prescription*"[tiab]
OR "Chinese herbal decoction*"[tiab]
OR "traditional Chinese herbal medicine*"[tiab]
OR "Chinese patent medicine*"[tiab]
OR "Chinese proprietary medicine*"[tiab]
OR "Chinese phytotherap*"[tiab]
OR "Chinese materia medica"[tiab]
OR "herb* medicine*"[tiab]
OR "herb* therap*"[tiab]
OR "herb* formula*"[tiab]
OR "herb* prescription*"[tiab]
OR "herb* decoction*"[tiab]
OR phytotherap*[tiab]
OR "materia medica"[tiab]
OR acupunct*[tiab]
OR electroacupunct*[tiab]
OR "scalp acupuncture"[tiab]
OR "head acupuncture"[tiab]
OR "auricular acupuncture"[tiab]
OR "ear acupuncture"[tiab]
OR "warm needl*"[tiab]
OR "fire needl*"[tiab]
OR moxibust*[tiab]
OR "warm moxibustion"[tiab]
OR "suspended moxibustion"[tiab]
OR tuina[tiab]
OR "Tui Na"[tiab]
OR "pediatric tuina"[tiab]
OR "Chinese massage"[tiab]
OR "traditional Chinese massage"[tiab]
OR cupping[tiab]
OR "cupping therapy"[tiab]
OR "wet cupping"[tiab]
OR "dry cupping"[tiab]
OR "fire cupping"[tiab]
OR "gua sha"[tiab]
OR guasha[tiab]
OR "scraping therapy"[tiab]
OR "acupoint* application*"[tiab]
OR "acupoint* sticking"[tiab]
OR "acupoint* plaster*"[tiab]
OR "acupoint* patch*"[tiab]
OR "acupoint* poultice*"[tiab]
OR "acupoint herbal application"[tiab]
OR "herbal patch*"[tiab]
OR "Chinese medical enema"[tiab]
OR "Chinese herbal enema"[tiab]
OR "herbal fumigation"[tiab]
OR "herbal bath*"[tiab]
)

#1 AND #2

Embase=456 (from inception to July 20, 2026)

#1

'cerebral palsy'/exp
OR (
'cerebral palsy'
OR 'cerebral palsies'
OR 'spastic diplegia'
OR 'spastic hemiplegia'
OR 'dyskinetic cerebral palsy'
OR 'athetoid cerebral palsy'
OR 'athetotic cerebral palsy'
OR 'mixed cerebral palsy'
):ti,ab,kw

#2

'traditional chinese medicine'/exp

OR 'chinese herbal medicine'/exp

OR 'acupuncture'/exp

OR 'electroacupuncture'/exp

OR 'moxibustion'/exp

OR (

'traditional chinese medicine'

OR 'chinese traditional medicine'

OR 'chinese medicine'

OR tcm

OR 'integrated chinese and western medicine'

OR 'integrated traditional chinese and western medicine'

OR 'integrative chinese medicine'

OR 'chinese herbal medicine*'

OR 'chinese herbal drug*'

OR 'chinese medicinal herb*'

OR 'chinese herbal formula*'

OR 'chinese herbal prescription*'

OR 'chinese herbal decoction*'

OR 'traditional chinese herbal medicine*'

OR 'chinese patent medicine*'

OR 'chinese proprietary medicine*'

OR 'chinese phytotherap*'

OR 'chinese materia medica'

OR phytotherap*

OR 'materia medica'

OR acupunct*

OR electroacupunct*

OR 'scalp acupuncture'

OR 'head acupuncture'

OR 'auricular acupuncture'

OR 'ear acupuncture'

OR 'warm needl*'

OR 'fire needl*'

OR moxibust*

OR 'warm moxibustion'

OR 'suspended moxibustion'

OR tuina

OR 'tui na'

OR 'pediatric tuina'

OR 'chinese massage'

OR 'traditional chinese massage'

OR cupping

OR 'cupping therapy'

OR 'wet cupping'

OR 'dry cupping'

OR 'fire cupping'

OR 'gua sha'

OR guasha

OR 'scraping therapy'

OR 'acupoint application'

OR 'acupoint application therapy'

OR 'acupoint sticking'

OR 'acupoint sticking therapy'

OR 'acupoint herbal application'

OR 'acupoint herbal patch*'

OR 'acupoint plaster*'

OR 'herbal patch*'

OR 'chinese medical enema'

OR 'chinese herbal enema'

OR 'herbal fumigation'

OR 'herbal bath*'

):ti,ab,kw

OR (

(herb* NEAR/3 medicine*)

OR (herb* NEAR/3 therap*)

OR (herb* NEAR/3 formula*)

OR (herb* NEAR/3 prescription*)

OR (herb* NEAR/3 decoction*)

):ti,ab,kw

#1 AND #2

CNKI=3328 (from inception to July 20, 2026)

(脑瘫 OR 脑性瘫痪)
AND
(
中医 OR 中医药 OR 中西医结合 OR 中医康复
OR 中药 OR 中草药 OR 草药 OR 中成药
OR 针刺 OR 针灸 OR 电针 OR 头针 OR 艾灸
OR 推拿 OR 按摩
OR 拔罐 OR 刮痧
OR 穴位贴敷 OR 中药外敷 OR 中药熏蒸
OR 中药熏洗 OR 中药药浴 OR 中药灌肠
OR 中医外治
)

Wanfang=3328 (from inception to July 20, 2026)

主题=(脑瘫 OR 脑性瘫痪)
AND
主题=(中医 OR 中医药 OR 中西医结合 OR 中医康复
OR 中药 OR 中草药 OR 草药 OR 中成药
OR 针刺 OR 针灸 OR 电针 OR 头针 OR 艾灸
OR 推拿 OR 按摩 OR 拔罐 OR 刮痧
OR 穴位贴敷 OR 中药外敷 OR 中药熏蒸
OR 中药熏洗 OR 中药药浴 OR 中药灌肠 OR 中医外治)

VIP=3839 (from inception to July 20, 2026)

篇关摘=(脑瘫 OR 脑性瘫痪)
AND
篇关摘=(中医 OR 中医药 OR 中西医结合 OR 中医康复
OR 中药 OR 中草药 OR 草药 OR 中成药
OR 针刺 OR 针灸 OR 电针 OR 头针 OR 艾灸
OR 推拿 OR 按摩 OR 拔罐 OR 刮痧
OR 穴位贴敷 OR 中药外敷 OR 中药熏蒸
OR 中药熏洗 OR 中药药浴 OR 中药灌肠 OR 中医外治)
