# Supplementary material for: Mapping traditional Chinese medicine-related complementary interventions for cerebral palsy rehabilitation: a bibliometric and visualization analysis
Source: Front Neurol. 2026 Aug 26;17:1882028. doi: 10.3389/fneur.2026.1882028 (PMC13553241; doi:10.3389/fneur.2026.1882028)
Supplement: Supplementary file 4 [file Table_2.DOCX]

**Supplementary Table S2. Software settings, analytical parameters, and reproducibility specifications**

| **Analysis component** | **Item** | **Chinese-language corpus** | **English-language corpus** | **Notes** |
| --- | --- | --- | --- | --- |
| General | Analytical corpus | 3,225 publications | 230 publications | The corpora were analyzed separately. |
| General | Descriptive analysis | Microsoft Excel 2019 | Microsoft Excel 2019 | Annual output and distributions of countries/regions, institutions, journals, and authors. |
| General | Keyword standardization | Corpus-specific merging and exclusion thesauri | Corpus-specific merging and exclusion thesauri | Two-reviewer checking after calibration; disagreements resolved by consensus. |
| VOSviewer | Software version and use | Version 1.6.20; keyword co-occurrence and overlay visualization | Version 1.6.20; keyword co-occurrence and overlay visualization | VOSviewer was not used for the final coauthorship analysis. |
| VOSviewer | Analysis type / unit | Co-occurrence / keywords | Co-occurrence / keywords | Author and indexed keywords were analyzed after cleaning. |
| VOSviewer | Counting method | Full counting | Full counting | Each keyword occurrence was counted in full. |
| VOSviewer | Normalization | Association-strength normalization | Association-strength normalization | Applied to keyword co-occurrence links. |
| VOSviewer | Standardized keywords before thresholding | 2,115 | 955 | After application of the corpus-specific thesauri. |
| VOSviewer | Principal minimum occurrence | 5 | 5 | 179 Chinese and 70 English keywords met the threshold. |
| VOSviewer | Minimum cluster size | 10 | 1 | Selected to accommodate the difference in network size and density. |
| VOSviewer | Layout parameters | Attraction = 10; repulsion = 6 | Attraction = 2; repulsion = 0 | The same corpus-specific settings were retained in the principal and occurrence-threshold sensitivity analyses. |
| VOSviewer | Clustering resolution | 1.0 | 1.0 | The identical setting was retained when occurrence thresholds were varied; insert the numeric resolution if it was manually changed. |
| VOSviewer | Node and link encoding | Node size = occurrences; link thickness = co-occurrence strength; color = cluster | Same | Clusters were generated automatically; descriptive labels were assigned subsequently. |
| VOSviewer | Overlay visualization | Same records, nodes, links, and threshold as the principal network | Same records, nodes, links, and threshold as the principal network | Node color represents average publication year. |
| VOSviewer | Overlay color scale | 2010–2020 | 2010–2020 | A common scale was used for visual comparison; values remain corpus-specific. |
| VOSviewer | Occurrence-threshold sensitivity | 3, 5 (principal), and 10 | 3, 5 (principal), and 7 | All other analytical settings were held constant; detailed results are reported in Supplementary Table S5. |
| CiteSpace | Software version and use | Version 6.4.2; keyword temporal and burst analysis | Version 6.4.2; keyword temporal and burst analysis | CiteSpace was not used for the co-occurrence maps. |
| CiteSpace | Analysis period | 1989–2025 | 2005–2025 | Earlier eligible records without sufficient indexable keywords remained in the complete bibliographic corpus. |
| CiteSpace | Time slicing | 1 year per slice | 1 year per slice | 2026 was excluded because the publication year was incomplete. |
| CiteSpace | Node type | Keyword | Keyword |  |
| CiteSpace | Node selection | g-index, k = 25 | g-index, k = 25 | No Top N or Top N% selection was used. |
| CiteSpace | Network pruning | None | None |  |
| CiteSpace | Burst states / alpha ratio | 2 states / 2.0 | 2 states / 2.0 | The alpha ratio is an occurrence-rate ratio, not a p value. |
| CiteSpace | Principal γ | 1.0 | 0.5 | Corpus-specific parameters were used because of the large difference in corpus size and temporal sparsity. |
| CiteSpace | Minimum burst duration | 2 years | 1 year |  |
| CiteSpace | Principal burst output | 39 keywords detected; top 25 displayed in Figure 5 | 18 keywords detected; all displayed in Figure 5 | Complete principal outputs are reported in Supplementary Table S4. |
| CiteSpace | Burst sensitivity analysis | Not performed | γ = 0.6/duration = 1 year; γ = 0.6/duration = 2 years | Detailed results are reported in Supplementary Table S6. |
| Coauthorship | Software | Python 3.13.5; NetworkX 3.6.1 | Python 3.13.5; NetworkX 3.6.1 | Microsoft Excel 2019 was used for descriptive author summaries. |
| Coauthorship | Records with author information | 3,222 of 3,225 | 229 of 230 | Author productivity was calculated using full counting. |
| Coauthorship | Network definition | Undirected, weighted, complete, and unthresholded | Undirected, weighted, complete, and unthresholded | Nodes = authors; edges = coauthorship; edge weight = number of joint publications; self-loops excluded. |
| Coauthorship | Degree centrality | Complete network; normalized by n − 1 | Complete network; normalized by n − 1 |  |
| Coauthorship | Betweenness centrality | Largest connected component; normalized unweighted shortest paths; endpoints excluded | Same |  |
| Coauthorship | Weighted collaboration strength | Sum of incident coauthorship-edge weights | Same |  |
| Coauthorship | Average clustering coefficient | Unweighted network | Unweighted network |  |
| Integrative synthesis | Analytical inputs | Co-occurrence, overlay, and burst results | Co-occurrence, overlay, and burst results | The framework was author-developed and not generated automatically by the software. |
| Integrative synthesis | Domain-retention rule | Support from at least 2 of 3 analytical outputs plus semantic coherence | Same | Two reviewers independently assessed candidate terms and domain labels; disagreements were resolved by consensus. |

*Notes: TLS, total link strength. Absolute node size, link strength, cluster membership, and burst strength were interpreted within each language-specific corpus and were not compared directly across corpora.*
