# Supplementary material for: Mapping traditional Chinese medicine-related complementary interventions for cerebral palsy rehabilitation: a bibliometric and visualization analysis
Source: Front Neurol. 2026 Aug 26;17:1882028. doi: 10.3389/fneur.2026.1882028 (PMC13553241; doi:10.3389/fneur.2026.1882028)
Supplement: Supplementary file 5 [file Table_3.DOCX]

**Supplementary Table S3. Leading authors according to coauthorship network centrality measures**

**Panel A. Chinese-language corpus: degree-centrality leaders**

| **Rank** | **Author** | **Publications** | **Unique coauthors** | **Weighted collaboration strength** | **Normalized degree centrality** | **Betweenness centrality (LCC)** |
| --- | --- | --- | --- | --- | --- | --- |
| 1 | Liu Zhenhuan (刘振寰) | 71 | 72 | 261 | 0.01311 | 0.11855 |
| 2 | Tai Xiantao (邰先桃) | 22 | 59 | 89 | 0.01074 | 0.08344 |
| 3 | Jin Bingxu (金炳旭) | 33 | 54 | 159 | 0.00983 | 0.13979 |
| 4 | Huang Mao (黄茂) | 25 | 49 | 86 | 0.00892 | 0.07113 |
| 5 | Zhao Yong (赵勇) | 30 | 48 | 128 | 0.00874 | 0.09070 |
| 6 | Wang Hui (王辉) | 20 | 42 | 69 | 0.00765 | 0.02088 |
| 7 | Shang Qing (尚清) | 26 | 41 | 60 | 0.00747 | 0.24523 |
| 8 | Zhou Yu (周钰) | 19 | 40 | 54 | 0.00728 | Not in LCC |
| 9 | Ma Bingxiang (马丙祥) | 32 | 39 | 82 | 0.00710 | 0.23439 |
| 10 | Li Nuo (李诺) | 20 | 35 | 98 | 0.00637 | 0.00161 |

**Panel B. Chinese-language corpus: betweenness-centrality leaders**

| **Rank** | **Author** | **Publications** | **Unique coauthors** | **Weighted collaboration strength** | **Normalized degree centrality** | **Betweenness centrality (LCC)** |
| --- | --- | --- | --- | --- | --- | --- |
| 1 | Zhu Min (朱敏) | 6 | 15 | 36 | 0.00273 | 0.35439 |
| 2 | Li Enyao (李恩耀) | 10 | 20 | 29 | 0.00364 | 0.35008 |
| 3 | Zhang Yong (张勇) | 9 | 20 | 31 | 0.00364 | 0.25859 |
| 4 | Shang Qing (尚清) | 26 | 41 | 60 | 0.00747 | 0.24523 |
| 5 | Zhai Hongyin (翟红印) | 7 | 13 | 23 | 0.00237 | 0.23733 |
| 6 | Ma Bingxiang (马丙祥) | 32 | 39 | 82 | 0.00710 | 0.23439 |
| 7 | Lou Yuanjun (娄元俊) | 10 | 24 | 41 | 0.00437 | 0.22926 |
| 8 | Tang Jian (汤健) | 12 | 21 | 49 | 0.00382 | 0.18994 |
| 9 | Gao Jing (高晶) | 24 | 34 | 83 | 0.00619 | 0.16154 |
| 10 | Yang Yang (杨阳) | 3 | 15 | 15 | 0.00273 | 0.14935 |

**Panel C. English-language corpus: degree-centrality leaders**

| **Rank** | **Author** | **Publications** | **Unique coauthors** | **Weighted collaboration strength** | **Normalized degree centrality** | **Betweenness centrality (LCC)** |
| --- | --- | --- | --- | --- | --- | --- |
| 1 | Liu, Y. | 11 | 65 | 75 | 0.08270 | 0.32812 |
| 2 | Wang, Y. | 5 | 49 | 52 | 0.06234 | 0.12504 |
| 3 | Zhao, Y. | 12 | 40 | 64 | 0.05089 | 0.28917 |
| 4 | Li, X. | 5 | 34 | 34 | 0.04326 | 0.06959 |
| 5 | Wu, Y. | 7 | 31 | 53 | 0.03944 | 0.29660 |
| 6 | Wang, J. | 5 | 30 | 31 | 0.03817 | 0.07642 |
| 7 | Li, M. | 3 | 29 | 32 | 0.03690 | 0.07278 |
| 8 | Liu, Z. H. | 15 | 28 | 69 | 0.03562 | 0.06148 |
| 9 | Zhang, Y. | 6 | 26 | 28 | 0.03308 | 0.07984 |
| 10 | Zhou, Y. | 5 | 25 | 27 | 0.03181 | 0.34913 |

**Panel D. English-language corpus: betweenness-centrality leaders**

| **Rank** | **Author** | **Publications** | **Unique coauthors** | **Weighted collaboration strength** | **Normalized degree centrality** | **Betweenness centrality (LCC)** |
| --- | --- | --- | --- | --- | --- | --- |
| 1 | Zhou, Y. | 5 | 25 | 27 | 0.03181 | 0.34913 |
| 2 | Liu, Y. | 11 | 65 | 75 | 0.08270 | 0.32812 |
| 3 | Wu, Y. | 7 | 31 | 53 | 0.03944 | 0.29660 |
| 4 | Zhao, Y. | 12 | 40 | 64 | 0.05089 | 0.28917 |
| 5 | Wang, H. | 2 | 19 | 19 | 0.02417 | 0.16870 |
| 6 | Zheng, H. | 6 | 19 | 46 | 0.02417 | 0.15981 |
| 7 | Zhang, J. | 5 | 20 | 20 | 0.02545 | 0.14904 |
| 8 | Wang, Y. | 5 | 49 | 52 | 0.06234 | 0.12504 |
| 9 | Yang, Y. | 3 | 20 | 20 | 0.02545 | 0.08795 |
| 10 | Huang, Y. | 2 | 17 | 17 | 0.02163 | 0.08331 |

*Notes: Degree centrality was calculated in the complete coauthorship network and normalized by n − 1. Betweenness centrality was calculated within the largest connected component using normalized, unweighted shortest paths with endpoints excluded. Weighted collaboration strength is the sum of the weights of all coauthorship edges incident to an author. Centrality describes structural position within the network and does not represent methodological quality or academic influence. Initial-based names in the English-language corpus and homonymous names in the Chinese-language corpus could not be fully disambiguated.*
