# Supplementary material for: Mapping traditional Chinese medicine-related complementary interventions for cerebral palsy rehabilitation: a bibliometric and visualization analysis
Source: Front Neurol. 2026 Aug 26;17:1882028. doi: 10.3389/fneur.2026.1882028 (PMC13553241; doi:10.3389/fneur.2026.1882028)
Supplement: Supplementary file 6 [file Table_4.DOCX]

**Supplementary Table S4. Complete keyword burst results in the Chinese- and English-language corpora**

**Panel A. Chinese-language corpus (1989–2025; γ = 1.0; minimum duration = 2 years)**

| **Original Chinese keyword** | **English display label** | **Year** | **Strength** | **Begin** | **End** | **Displayed in Figure 5** |
| --- | --- | --- | --- | --- | --- | --- |
| 头针 | scalp acupuncture | 1989 | 5.15 | 1989 | 2002 | Yes |
| 按摩 | massage | 1990 | 14.97 | 1990 | 2012 | Yes |
| 中西医结合治疗 | integrated traditional Chinese and Western medicine treatment | 1994 | 17.88 | 1994 | 2009 | Yes |
| 中医药治疗 | traditional Chinese medicine treatment | 1994 | 16.39 | 1994 | 2003 | Yes |
| 穴位注射 | acupoint injection | 1995 | 18.95 | 1995 | 2008 | Yes |
| 针刺疗法 | acupuncture | 1989 | 3.90 | 1996 | 1998 | No |
| 针灸 | acupuncture and moxibustion | 1991 | 4.31 | 1997 | 2000 | Yes |
| 中药治疗 | Chinese herbal medicine treatment | 1999 | 4.76 | 1999 | 2014 | Yes |
| 物理疗法 | physical therapy | 2001 | 4.20 | 2001 | 2005 | No |
| 高压氧 | hyperbaric oxygen therapy | 2001 | 4.09 | 2001 | 2010 | No |
| 上田法 | Ueda method | 2001 | 3.67 | 2001 | 2008 | No |
| 小儿 | children | 1997 | 32.67 | 2002 | 2006 | Yes |
| 体针 | body acupuncture | 1998 | 3.67 | 2002 | 2010 | No |
| 早期干预 | early intervention | 2000 | 5.98 | 2003 | 2014 | Yes |
| 中药 | Chinese herbal medicine | 2003 | 3.68 | 2003 | 2014 | No |
| 点穴疗法 | acupressure therapy | 2004 | 4.55 | 2004 | 2015 | Yes |
| 综述 | review | 2000 | 7.15 | 2005 | 2012 | Yes |
| 中药药浴 | Chinese herbal medicinal bath | 2005 | 5.66 | 2005 | 2012 | Yes |
| 姿势异常 | postural abnormality | 1997 | 3.71 | 2005 | 2009 | No |
| 中药熏蒸 | Chinese herbal fumigation | 2007 | 8.23 | 2007 | 2014 | Yes |
| 运动疗法 | exercise therapy | 1997 | 3.65 | 2007 | 2012 | No |
| 穴位疗法 | acupoint therapy | 1992 | 4.91 | 2010 | 2015 | Yes |
| 大鼠 | rat | 2000 | 3.73 | 2013 | 2016 | No |
| 粗大运动功能 | gross motor function | 2005 | 4.63 | 2014 | 2015 | Yes |
| 康复手法 | rehabilitation techniques | 2012 | 4.31 | 2014 | 2019 | No |
| 电针 | electroacupuncture | 1994 | 3.79 | 2014 | 2015 | No |
| 康复训练 | rehabilitation training | 1993 | 6.70 | 2017 | 2018 | Yes |
| 中医康复 | traditional Chinese medicine rehabilitation | 2006 | 4.28 | 2017 | 2022 | No |
| 针灸推拿 | acupuncture, moxibustion, and tuina | 2002 | 5.24 | 2018 | 2022 | Yes |
| 小儿推拿 | pediatric tuina | 1995 | 4.99 | 2018 | 2025 | Yes |
| 运动功能 | motor function | 2000 | 16.16 | 2019 | 2025 | Yes |
| 日常生活能力 | activities of daily living | 2002 | 6.86 | 2019 | 2023 | Yes |
| 运动发育推拿法 | motor-development tuina method | 2012 | 5.35 | 2019 | 2020 | Yes |
| 平衡功能 | balance function | 2007 | 5.87 | 2020 | 2025 | Yes |
| 肌张力 | muscle tone | 1992 | 5.76 | 2020 | 2022 | Yes |
| 数据挖掘 | data mining | 2020 | 3.71 | 2020 | 2025 | No |
| 运动功能障碍 | motor dysfunction | 1995 | 5.24 | 2021 | 2022 | Yes |
| 下肢运动功能 | lower-limb motor function | 2015 | 5.22 | 2021 | 2023 | Yes |
| 痉挛 | spasticity | 1992 | 3.90 | 2023 | 2025 | No |

**Panel B. English-language corpus (2005–2025; γ = 0.5; minimum duration = 1 year)**

| **Keyword** | **Year** | **Strength** | **Begin** | **End** |
| --- | --- | --- | --- | --- |
| clinical trial | 2005 | 3.28 | 2005 | 2011 |
| traditional Chinese medicine | 2005 | 2.07 | 2005 | 2005 |
| motor performance | 2005 | 2.07 | 2005 | 2005 |
| acupressure | 2005 | 1.66 | 2005 | 2006 |
| moxibustion | 2006 | 1.60 | 2006 | 2010 |
| controlled clinical trial | 2005 | 4.29 | 2007 | 2014 |
| infant | 2005 | 3.18 | 2010 | 2016 |
| pathophysiology | 2006 | 3.07 | 2010 | 2015 |
| acupuncture points | 2005 | 2.81 | 2010 | 2019 |
| motor activity | 2008 | 2.11 | 2010 | 2015 |
| acupuncture | 2005 | 1.67 | 2016 | 2017 |
| gross motor function measure | 2018 | 1.55 | 2018 | 2021 |
| ganglioside | 2019 | 1.71 | 2019 | 2021 |
| brain | 2007 | 2.07 | 2021 | 2023 |
| tuina | 2005 | 1.92 | 2021 | 2023 |
| spasticity | 2006 | 1.76 | 2021 | 2025 |
| scalp acupuncture | 2006 | 2.36 | 2022 | 2023 |
| cerebral palsy | 2005 | 1.57 | 2023 | 2025 |

*Notes: “Year” indicates the first year in which the keyword appeared in the analyzed corpus; “Begin” and “End” indicate the detected burst interval. Burst strength was interpreted within each corpus and was not compared directly between corpora. The Chinese main figure displays the 25 strongest of 39 detected bursts; all 18 English-language bursts are displayed. Chinese terms were translated using the predefined one-to-one display-label crosswalk, and the translation did not alter the burst calculations.*
