# Supplementary material for: Mapping traditional Chinese medicine-related complementary interventions for cerebral palsy rehabilitation: a bibliometric and visualization analysis
Source: Front Neurol. 2026 Aug 26;17:1882028. doi: 10.3389/fneur.2026.1882028 (PMC13553241; doi:10.3389/fneur.2026.1882028)
Supplement: Supplementary file 7 [file Table_5.DOCX]

Sensitivity analysis of VOSviewer minimum-occurrence thresholds

| **Corpus** | **Minimum occurrences** | **Minimum cluster size** | **Retained keywords** | **Links** | **Total link strength** | **Number of clusters** | **Leading keywords** | **Main thematic structure and parameter-related variation** |
| --- | --- | --- | --- | --- | --- | --- | --- | --- |
| Chinese-language | 3 | 10 | 331 | 3,342 | 13,909 | 16 | cerebral palsy; acupuncture; acupuncture and moxibustion; tuina; rehabilitation therapy | The principal themes involving acupuncture and moxibustion, tuina and other external TCM therapies, rehabilitation and functional outcomes, cerebral-palsy subtypes and symptoms, herbal treatments, and mechanistic research were retained. Additional low-frequency techniques, syndromes, rehabilitation modalities, and mechanistic terms produced a more granular and fragmented thematic structure. |
| Chinese-language | **5 (principal)** | 10 | 179 | 2,415 | 12,505 | 10 | cerebral palsy; acupuncture; acupuncture and moxibustion; tuina; rehabilitation therapy | The network provided a balanced representation of acupuncture-related interventions, tuina and external TCM therapies, rehabilitation and functional outcomes, clinical manifestations and subtypes, and mechanistic research. |
| Chinese-language | 10 | 10 | 110 | 1,728 | 11,336 | 6 | cerebral palsy; acupuncture; acupuncture and moxibustion; tuina; rehabilitation therapy | The principal high-frequency themes remained identifiable, but lower-frequency treatment techniques and mechanistic subthemes were reduced or incorporated into broader clusters. |
| English-language | 3 | 1 | 122 | 1,993 | 5,013 | 5 | cerebral palsy; acupuncture; randomized controlled trial; preschool child; infant | Five broad themes were retained, covering animal and mechanistic research, clinical evaluation and motor outcomes, acupuncture-related pediatric research, TCM and rehabilitation, and tuina or scalp-acupuncture applications. The lower threshold increased the visibility of low-frequency methodological and mechanistic terms. |
| English-language | **5 (principal)** | 1 | 70 | 1,045 | 3,587 | 5 | cerebral palsy; acupuncture; randomized controlled trial; preschool child; infant | The network provided a balanced representation of clinical study design, pediatric acupuncture research, TCM-related interventions, motor-function outcomes, rehabilitation, evidence synthesis, and experimental mechanisms. |
| English-language | 7 | 1 | 43 | 508 | 2,575 | 4 | cerebral palsy; acupuncture; randomized controlled trial; preschool child; infant | Four coherent themes remained visible: clinical TCM interventions and motor outcomes; animal and mechanistic research; acupuncture-related pediatric clinical research; and rehabilitation, spasticity, and evidence synthesis. Peripheral low-frequency terms were reduced without eliminating the principal thematic structure. |
