# Supplementary material for: Mapping traditional Chinese medicine-related complementary interventions for cerebral palsy rehabilitation: a bibliometric and visualization analysis
Source: Front Neurol. 2026 Aug 26;17:1882028. doi: 10.3389/fneur.2026.1882028 (PMC13553241; doi:10.3389/fneur.2026.1882028)
Supplement: Supplementary file 8 [file Table_6.DOCX]

Sensitivity analysis of burst-detection parameters in the English-language corpus

| **γ** | **Minimum burst duration** | **Detected burst keywords** | **Strongest burst terms** | **Recent burst terms beginning in or after 2021** | **Terms active through 2025** | **Interpretation** |
| --- | --- | --- | --- | --- | --- | --- |
| 0.5 | 1 year | 18 | controlled clinical trial (4.29); clinical trial (3.28); infant (3.18) | brain; tuina; spasticity; scalp acupuncture; cerebral palsy | spasticity; cerebral palsy | Principal, more inclusive setting |
| 0.6 | 1 year | 11 | controlled clinical trial (4.29); clinical trial (3.28); infant (3.18) | brain; tuina; scalp acupuncture | None | Stronger subset retained; weaker recent bursts were removed |
| 0.6 | 2 years | 3 | controlled clinical trial (4.29); clinical trial (3.28); infant (3.18) | None | None | Only the most sustained early bursts were retained |
